# PRC2 inhibition counteracts the culture-associated loss of engraftment potential of human cord blood-derived hematopoietic stem and progenitor cells

**DOI:** 10.1038/srep12319

**Published:** 2015-07-22

**Authors:** Linda Varagnolo, Qiong Lin, Nadine Obier, Christoph Plass, Johannes Dietl, Martin Zenke, Rainer Claus, Albrecht M. Müller

**Affiliations:** 1 Institute of Medical Radiology and Cell Research (MSZ) in the Center for Experimental Molecular Medicine (ZEMM), University of Würzburg, Würzburg, Germany; 2 Department of Cell Biology, Helmholtz Institute for Biomedical Engineering, RWTH Aachen University, Aachen, Germany; 3 School of Cancer Sciences, University of Birmingham, Birmingham, United Kingdom; 4 Department of Epigenomics and Cancer Risk Factors, German Cancer Research Center (DKFZ), Heidelberg, Germany; 5 Department of Gynecology and Obstetrics, Medical University of Würzburg, Germany; 6 Department of Medicine, Div. Hematology, Oncology and Stem Cell Transplantation, University of Freiburg Medical Center, Freiburg, Germany

## Abstract

Cord blood hematopoietic stem cells (CB-HSCs) are an outstanding source for transplantation approaches. However, the amount of cells per donor is limited and culture expansion of CB-HSCs is accompanied by a loss of engraftment potential. In order to analyze the molecular mechanisms leading to this impaired potential we profiled global and local epigenotypes during the expansion of human CB hematopoietic stem and progenitor cells (HPSCs). Human CB-derived CD34+ cells were cultured in serum-free medium together with SCF, TPO, FGF, with or without Igfbp2 and Angptl5 (STF/STFIA cocktails). As compared to the STF cocktail, the STFIA cocktail maintains *in vivo* repopulation capacity of cultured CD34+ cells. Upon expansion, CD34+ cells genome-wide remodel their epigenotype and depending on the cytokine cocktail, cells show different H3K4me3 and H3K27me3 levels. Expanding cells without Igfbp2 and Angptl5 leads to higher global H3K27me3 levels. ChIPseq analyses reveal a cytokine cocktail-dependent redistribution of H3K27me3 profiles. Inhibition of the PRC2 component EZH2 counteracts the culture-associated loss of NOD scid gamma (NSG) engraftment potential. Collectively, our data reveal chromatin dynamics that underlie the culture-associated loss of engraftment potential. We identify PRC2 component EZH2 as being involved in the loss of engraftment potential during the *in vitro* expansion of HPSCs.

Hematopoietic stem cells (HSCs) are a rare cell type that are essential for life-long blood production. The transplantation of HSCs has evolved from a highly experimental procedure to a standard therapy for several malignant and non-malignant hematologic and other diseases^1^. Today, most HSC transplant samples are isolated from peripheral blood after mobilization or from bone marrow (BM) aspirates of healthy donors. Cord blood (CB)-derived HSCs are a third source of HSCs for patients with hematologic disorders and metabolic storage diseases^2^. CB-HSC transplantation is increasingly used because of its availability, banking features and lower incidence of severe chronic graft-versus-host disease (GvHD) leading to reduced HLA-requirement compared to BM cells. However, limited cell numbers per isolate restrict CB transplantation. Despite optimization of isolation and processing techniques, the low cell numbers per isolate and the inability to robustly expand CB-HSCs renders insufficient stem cell numbers a major constraint in many transplantation settings. One approach to overcome the low cell content of single CB units is co-transplantation of two units^3^.

A multitude of cell-intrinsic and extrinsic self-renewal factors and combinations thereof in addition to stromal cell cultures were assessed for their ability to robustly expand HSCs^4^,^5^. Proliferation of HSCs could be achieved by *in vitro* cultures but often stem cell properties such as longterm and multlineage engraftment were lost. While transcriptome studies of HSCs did so far not lead to novel concepts of HSC expansion^6^,^7^, other studies explored the cytokine profile of murine HSC supporter cells and the HSC receptor status in fetal liver, the developmental stage and physiological side of high HSC expansion^8^. This approach introduced Insulin-like growth factor-binding protein 2 (Igfbp2) and a group of angiopoietin-like (Angptl) proteins, secreted glycoproteins consisting of seven members, as alternative growth factors for HSCs expansion^9^.

The self-renewal and differentiation of HSCs is linked to interconnected transcriptional and epigenetic circuits, both triggered by extra- and intracellular signals^10^. Epigenetic mechanisms directly shape and progressively restrict the lineage potential of HSCs by controlling chromatin compaction and accessibility^11^,^12^. Particularly, the evolutionary conserved Polycomb-group (PcG) and Trithorax-group (trxG) proteins play pivotal roles in the regulation of HSC function^13^,^14^. Both act as multifactorial complexes that influence gene expression by adding specific modifications to histone tails. While the Polycomb repressive complex (PRC) 2 silences genes by tri-methylation of histone H3 lysine 27 (H3K27), trxG proteins act antagonistically *via* the generation of H3K4me3 marks^15^. The simultaneous marking of genes with activating H3K4me3 and repressive H3K27me3 modifications (bivalent domains) poises chromatin for activation^16^. Remodelling of the bivalent landscape accompanies the differentiation of HSCs^12^,^17^,^18^. Maps of the epigenetic landscapes of HSCs and differentiated progeny revealed that combinatorial modification patterns ensure cooperative regulation of transcription supporting the notion that epigenetics accompanies HSC function and differentiation^17^. This notion is increasingly translated into practice as epigenetic strategies are considered for HSC expansion and as treatment option of hematopoietic malignancies^19^, ^20^,^21^.

While high-resolution and genome-wide histone modification maps of fresh mouse and human HSCs were described^12^,^17^,^22^, it largely remains open how *ex vivo* culture conditions influence chromatin modifications of HSCs. Upon culture expansion of human CB-CD34+ hematopoietic progenitor/stem cells (HPSCs) were shown to acquire DNA-hypermethylation at specific sites in the genome^23^,^24^.

Here, we assessed epigenetic changes in fresh and culture-expanded CB-HPSCs. We aimed at identifying epigenetic target mechanisms associated with *ex vivo* expansion. In summary, we show that culture expansion induced global and local changes of H3K4me3 and H3K27me3 profiles. We detected widespread variations of global H3K27me3 levels and genome-wide redistribution that are connected to elevated expression of the PRC2 component EZH2. Inhibition by EZH2 inhibitor treatment increased engraftment potential of cultured HPSCs.

## Materials and Methods

### Isolation and culture of CB-derived hematopoietic progenitor/stem cells

Human CB cells were isolated from fresh CB samples obtained from the Department for Gynecology and Obstetrics, University Clinic Würzburg, after written consent according to the guidelines specifically approved by the Ethic Committee of the Medical Faculty of the University of Würzburg, Germany (Permit Number: 185/08). CD34+ progenitors were obtained using a CD34 progenitor cell isolation kit (Miltenyi Biotec). The purities of CD34+ samples were determined *via* flow cytometry. Immunophenotype of CD34+ cells were: 91% CD34+; 86.9% CD133+; 33% CD38+; 98% CD45+; immunophenotype of CD34− cells were: 1% CD34+; 1.2% CD133+; 46.8% CD38+; 58% CD45+. For expansion, CD34+ progenitor cells were seeded at a concentration of 2 × 10^5^ cells/ml into StemSpan serum-free medium (StemCell Technologies Inc.) supplemented with 1% penicillin, streptomycin (Gibco Invitrogen Corporation), heparin (10 ng/mL, Ratiopharm) plus either STF (stem cell factor (SCF, 10 ng/mL, Peprotech), recombinant human thrombopoetin (TPO, 20 ng/mL, Peprotech), recombinant human fibroblast growth factor-1 (FGF-1, 10 ng/mL, Peprotech)), or STFIA (STF plus angiopoietin like-5 (Angptl-5, 500 ng/mL, Tebu-Bio) and insulin-like growth factor binding-protein 2 (IGFBP2, 100 ng/mL, R&D Systems)) cocktails^9^. CD34+ cells in STF culture were co-incubated with either GSK343 (SGC, University of Toronto) or GSK126 EZH2 inhibitors (Chemie Tek) at 1 mM.

### NSG transplantation

NOD.Cg-Prkdcscid Il2rgtm1Wjl/SzJ (NSG) mice were bred and maintained at the ZEMM (University Würzburg, Germany). All animals handling was done according to the animal protection guidelines of the government of Unterfranken, Germany. Fresh or expanded cells were injected intravenously into sub-lethally irradiated (1.5 Gy, Faxitron CP-160 X-ray radiation cabinet (160 kV, 6.3 mA, 0.4 Gray/min, filter: 0.5 mm Cu)) 8–10 weeks old female NSG recipients. 0.5 × 10^5^ fresh CD34+ cells together with 10^5^ NSG spenocytes were injected per recipient. The progeny of 2 × 10^5^ CD34+ cells (injected cell numbers per animal ranged from 0.4–1 × 10^6^ cells) expanded in STF or STFIA cultures were equally split and injected into 4 recipients. Multilineage engraftment was analyzed by staining peripheral blood, splenocytes and BM of recipients with human antibodies specific for CD45, CD19, CD14, CD3 or CD34 or with matched isotype controls (Miltenyi Biotec). Secondary transplantations were carried out at 16 weeks post-primary transplantation. The BM cells of both tibias and femurs of primary recipients were injected into two sub-lethally irradiated secondary recipients.

### Flow cytometry and chromatin flow cytometry

Cells were incubated with FITC- or PE-labeled CD45−, CD34−, CD133−, CD38−, CD14−, CD19−, CD3-specific and matching isotype antibodies (Miltenyi Biotec). For chromatin flow cytometry^25^, cells were added dropwise into tubes containing 1 ml ice-cold 88% MeOH/PBS for 30 minutes. Primary antibodies (H3K4me3 (Abcam), H3K27me3 (Diagenode), H3K79me3 (Abcam) and H3K9me3 (Upstate)) were added and samples were incubated (1 h, 4 °C). For normalization, cells were stained with an H3-specific antibody (Abcam). Cells were analyzed using a FACS Canto machine (BD) running FACS Diva software (BD).

### Western blot analyses

Protein samples were prepared and blotted onto nitrocellulose membranes after blocking unspecific binding sites. Membranes were incubated at 4 °C over night with primary antibodies (anti-H3K4me3 (Abcam), anti-H3K27me3 (Diagenode), anti-H3 (Abcam), anti-EZH2 (Diagenode)). Following 3 washes, membranes were incubated (2 h, RT) with secondary HRP-coupled antibodies, before proteins were detected by ECL addition (ECL, Amersham Biosciences) and chemiluminescence measurement.

### ChIPseq analyses

ChIPseq analyses were conducted as replicates, merged and analyzed following guidelines and recommendations of the ENCODE consortium^26^. For detailed description see Supplemental Material.

### Statistical analysis

Results are indicated with standard error of the average of independent experiments. Analyses were done by two-tailed Student t-test. Probability value of p < 0.05 denoted statistical significance.

## Results

### Expansion of CD34+ cells is accompanied by global histone modification changes

**Figure 1:**
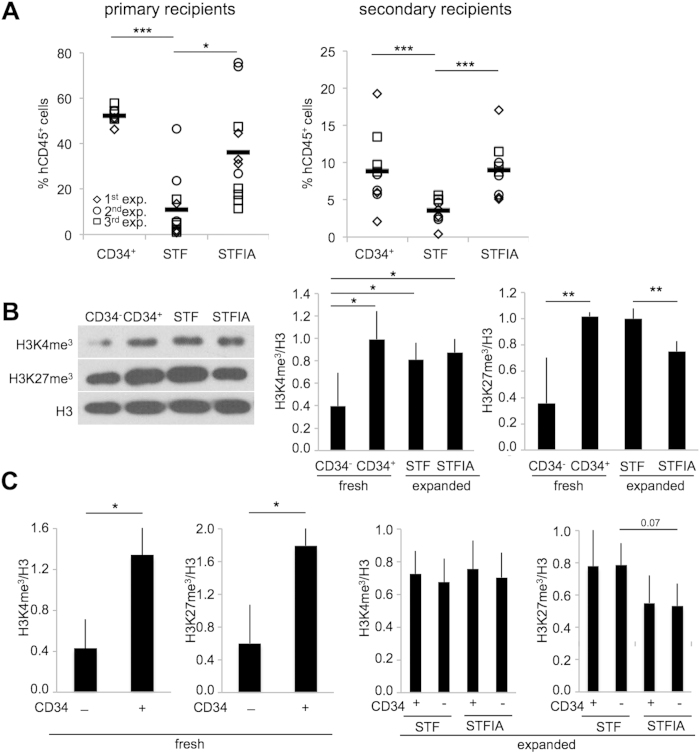
Engraftment activities and global H3K27me3 and of H3K4me3 levels in fresh and expanded CD34+ cells. **A**) Shown are percentages of human chimerism by CD45-specific flow cytometry in the bone marrow of NOD-scid IL2Rγnull (NSG) transplant recipients. After 7 days of culture the progeny of 2 × 10^5^ CD34+ cells in STF- or STFIA-supplemented cultures were equally split and injected into 4 recipients. Recipients injected with 0.5 × 10^5^ fresh CD34+ cells are shown as controls. Each symbol represents the engraftment of a single recipient (left). Also shown are percentages of human chimerism in the bone marrow of mice following transplantation of bone marrow cells from primary into secondary recipients (right). The bone marrow of one primary was injected into two secondary recipients. Recipients were analyzed 8 weeks post transplant. Student t-test, ***p < 0.001, *p < 0.05 n = 3. **B**) Representative H3K4me3- and H3K27me3-specific Western blot analysis of fresh CD34− and CD34+ cells, and of CD34+ cells expanded for 7 days with either STF or STFIA cocktails (left). Relative signal intensities of bands were quantified (middle, right). n = 4. Student t-test, **p < 0.01, *p < 0.05. **C**) Summary of H3-normalized values of global H3K4me3 and H3K27me3 fluorescence levels of fresh CD34− and CD34+ cells, and of CD34+ cells expanded for 7 days with either STF or STFIA cocktails analyzed via chromatin flow cytometry, *p < 0.05, n = 4.

We sought to determine whether and how the expansion of CB-HPSCs genome-wide alters histone modifications and remodels global and local chromatin states. First, functional characterization of CD34+ cells cultured under different cytokine cocktail conditions confirmed the increased multilineage engraftment potential in primary and secondary NSG recipients in STFIA- versus STF-cultured CB CD34+ cells (Fig. 1A, Fig. S1)^9^. Notably, the CD34+ fraction is heterogeneous and only a small subset resembles hematopoietic stem cells (HSCs). Global gene expression analyses of STF- versus STFIA-cultured CD34+ cells and cluster analysis indicated no statistically significant differences between STF and STFIA samples (p-value < 0.05 and fold change >2) and that samples from one donor clustered together (Fig. S2A). This is underlined by the heatmaps of differentially expressed genes that revealed only minor transcriptomic changes between STF- versus STFIA-cultured CD34+ samples while gene expression between fresh and expanded CD34+ cells differed (Fig. S2B). Genes that were down-regulated upon STF or STFIA expansion belonged to Gene Ontology (GO) categories ‘regulation of transcription’ and ‘cell death’, while genes that were up-regulated during culture were enriched for GO terms associated with cell cycle regulation (Table S3, Fig. S3). To assess culture-induced global epigenetic changes we used Western blot and intranuclear flow cytometry^25^. Both analyses revealed higher levels of the activating H3K4me3 and the repressive H3K27me3 modifications in fresh CD34+ compared to CD34− cells. Upon expansion of CD34+ cells, we noticed that global H3K4me3 levels did not differ significantly between STF and STFIA cultures whereas STFIA-cultured cells carried less H3K27me3 than fresh CD34+ or STF-cultured CD34+ cells (Fig. 1B,C). Despite similarities in gene expression between the two different culture conditions, just the STFIA-cultured cells showed elevated repopulation potential. Together this shows that changes in H3K27me3 levels but less changes in H3K4me3 levels accompany the culture-associated loss of engraftment potential.

### Redistribution of K4 and K27 tri-methylation marks upon culture of CD34+ cells

**Figure 2:**
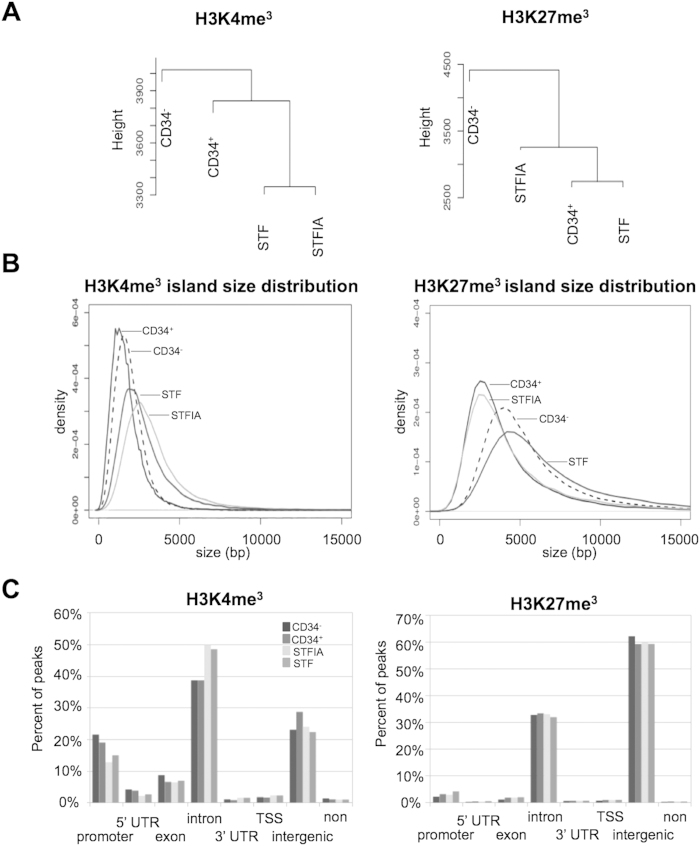
Genome-wide distribution of H3K4me3 and H3K27me3 profiles in fresh and expanded CD34+ cells. **A**) Unsupervised hierarchical cluster analysis using Ward’s minimum variance of H3K4 and H3K27 trimethylation ChIPseq data sets originating from fresh CD34− and CD34+ cells, and from CD34+ cells expanded for 7 days with either STF or STFIA cocktails. Data sets of two independent experiments per sample were merged. The entire sequenced genome was partitioned in 500 bp bins. **B**) H3K4me3 and H3K27me3 peak size distributions in fresh CD34− and CD34+ cells, and in CD34+ cells expanded for 7 days with either STF or STFIA cocktails using kernel density graphs. Peaks were called using the using SICER V1.1. **C**) Genome-wide distribution of H3K4me3 and H3K27me3 histone modifications. Shown are percentages of peaks in promoter, 5′UTR, exon, intron, 3′UTR, TSS, intergenic and non-intergenic regions.

To characterize the impact of *in vitro* expansion on chromatin signatures in greater detail, we performed H3K4me3- and H3K27me3-specific chromatin immunoprecipitation and next generation sequencing (ChIPseq) and mapped histone modifications associated with active and repressed transcriptional states on a genome-wide level. Procedures were optimized for low cell numbers^27^. Typically 80,000 cells were used for ChIPseq analysis. Following the experimental standards as set by the ENCODE consortium, replicates were processed independently and merged for subsequent data analysis, as the number of ChIPseq detected sites typically increases with the number of sequencing reads and, particularly, weaker sites can be detected with greater confidence in deeper data sets because of the increased statistical power^28^. Raw data processing and quality control revealed high replicate correlations between individual samples according to ENCODE ChIPseq guidelines (Fig. S4)^26^. To assess similarities between individual samples, we firstly performed unsupervised hierarchical clustering of complete data sets comprising the entire genome partitioned into 500 bp bins. This clustering using Ward’s minimum variance method revealed that H3K4me3 samples showed a closer relationship between the datasets of the two expanded compared to fresh cells (Fig. 2A). In contrast, the H3K27me3 samples of STF- and STFIA-cultured and fresh CD34+ cells grouped together while the CD34− sample separated. A closer inspection revealed that H3K4me3 meta-profiles on all promoters and gene bodies were maintained during expansion of CD34+ cells, independently of cocktail composition while fresh CD34− cells exhibited a slightly higher ChIPseq read density (Fig. S5A). Upon expansion of CD34+ cells, H3K27me3 levels at transcriptional start sites and within gene bodies decreased.

We then used peak detection to identify regions of significant H3K4me3 and H3K27me3 enrichment. Overall, we noticed that larger portions of the genome were enriched with H3K27me3 compared to H3K4me3 marks (Fig. S5B). The genome fraction covered by H3K4me3 enrichment (peaks) was larger in STFIA- and STF-cultured CD34+ as compared to fresh cells. In contrast, the genome fraction covered by H3K27me3 peaks was increased upon STFIA- but not STF-culturing. Peak counts of H3K27me3-marked regions in STF cells were reduced compared to other samples. Analyzing the size of H3K4me3- and H3K27me3-enriched regions (islands, horizontal expansion) we observed that H3K4me3 regions in fresh CD34− and CD34+ cells were similarly sized, while STF or STFIA culture increased the size of H3K4me3-marked regions (Fig. 2B). In parallel with the reduced peak counts of H3K27me3-marked regions in STF-cultured cells, H3K27me3-enriched regions were considerably broadened in STF-cultured cells similar to fresh CD34− cells.

Genome-wide analysis of H3K4me3 and H3K27me3 peak distribution over functional genomic elements revealed that promoters, introns and intergenic regions were preferentially H3K4me3-marked. In contrast, H3K27me3 was widely deposited at introns and intergenic regions (Fig. 2C). Upon expansion of CD34+ cells, promoters lost and introns gained H3K4me3. Culture conditions had no visible qualitative or quantitative impact on the lawn of H3K27me3 marking at the analyzed elements.

Next, we asked if the average profiles of the H3K4me3 and the H3K27me3 marks differ at the transcriptional start sites of those 2,000 genes which are expressed highest and lowest in fresh CD34+ cells (Fig. S6A). The analysis revealed a typical distribution of H3K4me3 with a nucleosome gap at the transcriptional start site and almost complete lack of H3K27me3 on active promoters for the highly expressed genes. For these 2,000 genes highest expressed in CD34+ cells, STFIA-cultured CD34+ cells exhibited an almost identical H3K4me3 promoter profile as fresh CD34+ cells, whereas STF-cultured CD34+ cells and CD34− cells had higher and lower tag density, respectively. Correspondingly, the lowest expressed genes carried almost no H3K4me3 and more repressive H3K27me3 marks in all 4 samples (Fig. S6B).

**Figure 3:**
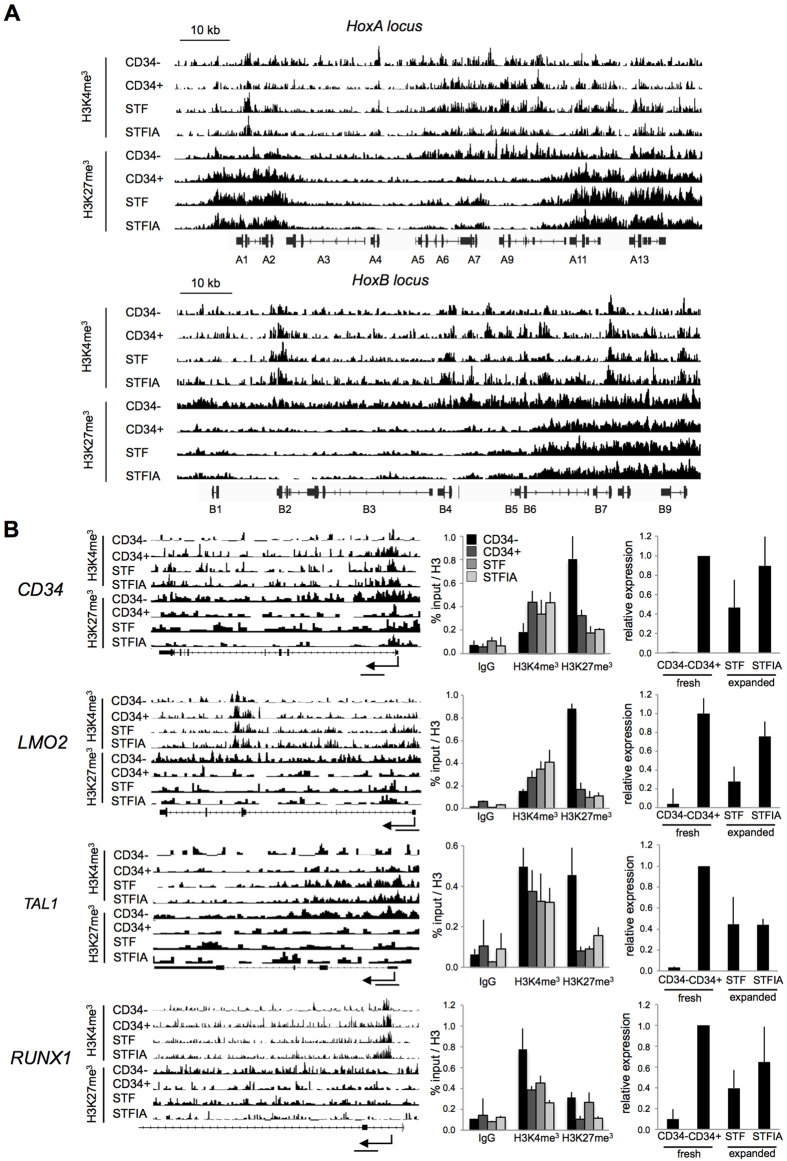
Culture-induced histone modification changes. **A**) Shown are H3K4me3- and H3K27me3 profiles in the HoxA (chr7:27,132,263 – 27,246,155) and HoxB (chr17:46,606,551 – 46,703,627) loci in fresh CD34− and CD34+ cells, and in CD34+ cells expanded for 7 days with either STF or STFIA cocktails. Data are displayed using the Integrative Genomics Viewer software. The positions of HoxA and HoxB genes are indicated. On the y-axis the number of reads per 200 bp windows are displayed. The position of introns, exons and the direction of transcription are shown. **B**) ChIP-seq profiles, ChIP-PCR and RT-PCR analyses of selected genes in fresh CD34− and CD34+ cells and in STF- and STFIA-expanded cells. The TSS and the amplicon location relative to the TSS are indicated. Sizes of entire loci were: CD34, 31 kb; LMO2, 37 kb; TAL1, 17 kb; RUNX1, 95 kb.

Previously, Cui *et al.* reported broad changes of H3K4me3 and H3K27me3 patterns in homeobox (Hox) gene clusters during CB-HSC differentiation^17^. Therefore we assessed whether culture of CD34+ cells is paralleled by changes in H3K4me3 and H3K27me3 marking in the HoxA and HoxB clusters (Fig. 3A). Overall we noticed differences in H3K27me3 deposition at the HoxA and HoxB loci between fresh CD34+ and CD34− cells but little changes between the culture conditions. Less differences in H3K4me3 were visible between fresh and cultured cells. Overall, expansion of CD34+ cells maintained the distribution of H3K4me3 and H3K27me3 patterns in HoxA and HoxB loci as seen in fresh cells with only minor differences (i.e. in HoxA5 - A9 regions). H3K4me3- and H3K27me3-specific ChIP for selected promoter regions of development- and HPSC-specific genes were employed for further characterization of ChIP-seq profiles (Fig. 3B). Overall it appears that the distribution of H3K4me3 and H3K27me3 in ChIPseq marks was consistant with ChIP-PCR analysis in promoter regions and corresponded to the transcriptional states of the selected genes. Depending on the locus, H3K27me3 patterns changed between the two culture conditions more than H3K4me3 patterns. For example, in the CD34 gene body, STF-cultured cells showed higher enrichment of H3K27me3 compared to STFIA cells while H3K4me3 patterns were similar.

**Figure 4:**
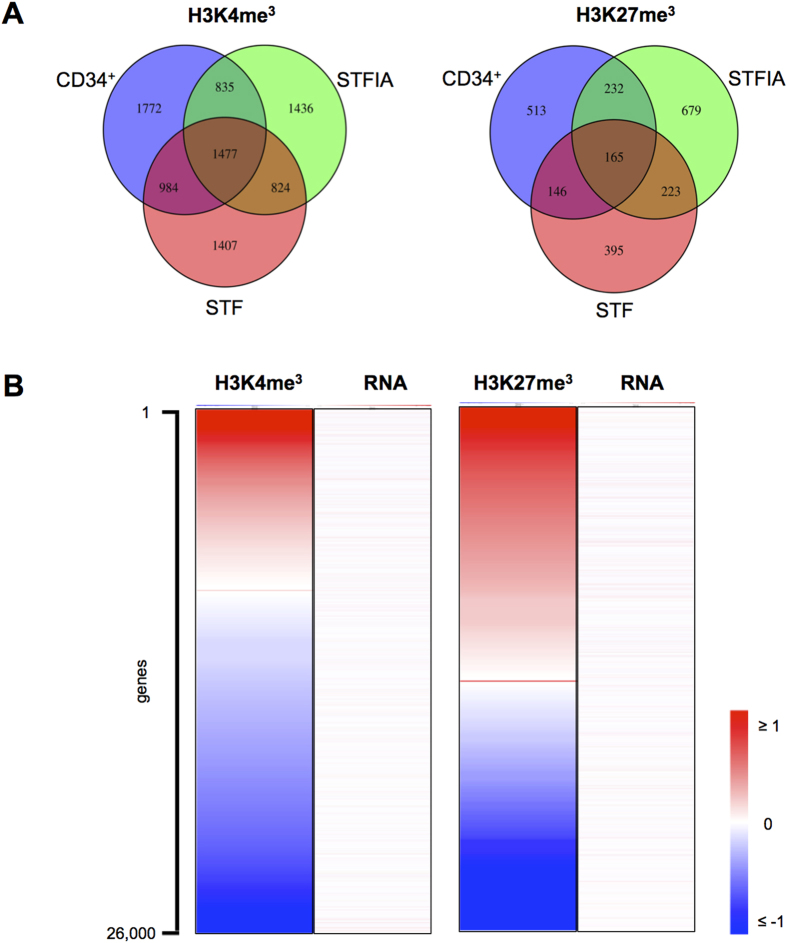
Analysis of promoters enriched for H3K4me3 or H3K27me3. **A**) Venn diagrams show numbers of promoters marked with either H3K4me3 or H3K27me3 in fresh CD34+ cells, and in CD34+ cells expanded for 7 days with either STF or STFIA cocktails. (n = 2). **B**) Approximately 26,000 transcripts taken from the Affymetrix expression arrays of STFIA- and STF-cultured CD34 + cells were ranked according to the log2-fold change (STFIA *versus* STF) of sequencing reads in their promoter regions (defined as –1000 bp to +500 bp around transcriptional start site). Log2-fold changes ranged from –3.84 to 4.02 for H3K4me3 and –4.19 to 3.51 for H3K27me3 and were displayed by color coding (shades of red = positive log2-fold changes, grey = no change/0, blue = negative log2-fold changes). The same representation was chosen for the log2-fold changed mRNA expression values for the respective transcripts taken from the Affymetric expression arrays (lane labelled ‚RNA’).

A closer look at H3K4me3- or H3K27me3-enriched promoters revealed that culture conditions changed the set of enriched promoters in an individual way (Fig. 4A, Table S1). One third of all promoters enriched with either H3K4me3 or H3K27me3 were exclusively marked depending on whether fresh, STF- or STFIA-cultured cells were analyzed. GO analysis of H3K4me3-enriched promoters mostly revealed functional categories of ‘transcriptional regulation’ and ‘metabolic processes’ in fresh and STFIA-expanded CD34+ cells while STF-expanded cells associated with ‘signaling’ and ‘cell death’ (Fig. S7). The H3K27me3-enriched promoters were associated with the GO-terms ‘signal transduction’ and ‘differentiation’ in fresh and cultured samples.

To assess the relation between chromatin modification changes and altered gene expression, we ranked 26,000 genes according to the fold-change of H3K4me3 or H3K27me3 sequencing reads in their promoter regions and asked for changes in gene expression. In a direct comparison of STFIA *versus* STF culture conditions, we noticed more H3K4me3 losses than gains (Fig. 4B). While H3K4me3 changes appeared to be more balanced with similar extent of lossed and gained areas, the extent of H3K27me3 changes was higher compared to H3K4me3 changes (as indicated by the enlarged saturated red and blue areas). The greater extent of H3K27me3 changes compared to H3K4me3 was also shown by the larger area below the line plot of H3K27me3 (Fig. S8A). This indicates a higher remodelling of H3K27me3-enriched promoters compared to H3K4me3 promoters without transcriptomic changes. Under both culture conditions, gain of H3K27me3 at promoter regions was more frequent than loss when compared to fresh CD34+ cells (Fig. S8B, C). In contrast, H3K4me3 changes were almost balanced in STFIA-cultured CD34+ cells whereas H3K4me3 gain was predominant upon STF culture compared to fresh CD34+ cells. For neither culture condition we observed a correlation between H3K4me3 or H3K27me3 changes and gene expression.

**Figure 5:**
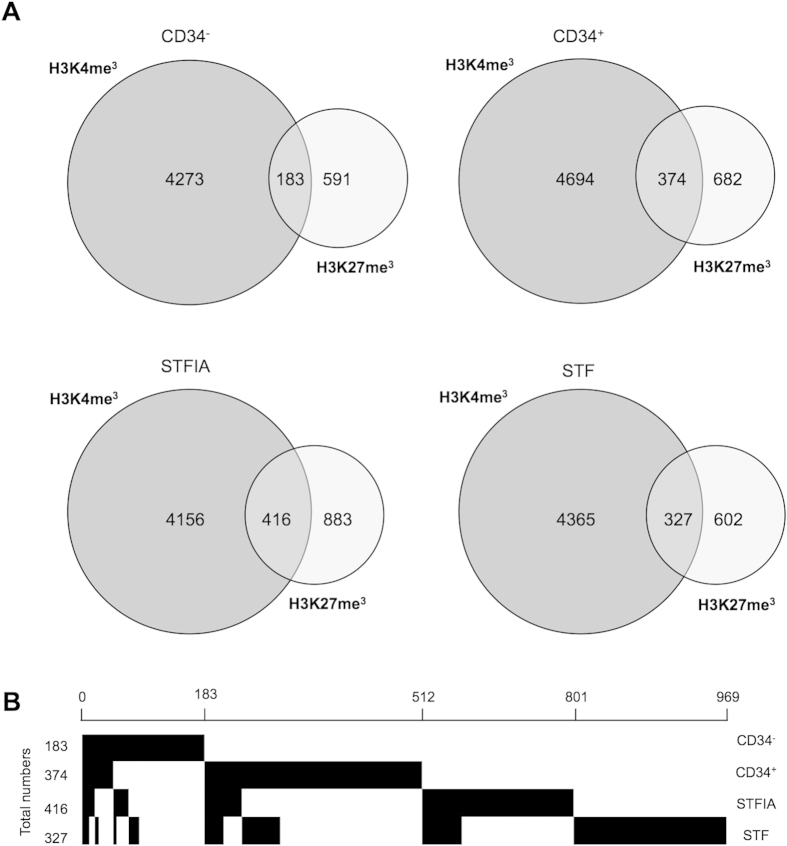
Co-occupancy of H3K4me3 and H3K27me3 on promoters. **A**) Numbers of promoters marked with H3K4me3 and H3K27me3 and numbers of H3K4me3 and H3K27me3 co-occupancy on promoters in fresh CD34− and CD34+ cells, and in CD34+ cells expanded for 7 days with either STF or STFIA cocktails. **B**) H3K4me3 and H3K27me3 co-occupancy on promoters and patterns of recurrence and mutual exclusion in fresh and cultured cells. Each column represent a gene recorded as bivalent in any of the 4 samples.

Finally, we looked at the number of promoters with both H3K4me3 and H3K27me3 marks and noticed that fresh CD34+ and STF- or STFIA-cultured cells showed twice the numbers of promoters with the bivalent mark compared to CD34− cells (Fig. 5A). To assess the dynamics of co-occupancy resolution upon expansion we constructed maps of promoter bivalency status of fresh and cultured cells (Fig. 5B). Only about one third of the genes marked with both modifications in fresh CD34+ cells maintained the bivalent mark upon STF or STFIA expansion. GO analysis revealed enrichment of genes belonging to blood vessel development and morphogenesis in STFIA samples (Fig. S9, Table S2). Overall, we observed a cocktail-specific reorganization of the bivalency status.

### EZH2 inhibition increases engrafting potential of cultured CD34+ cells

**Figure 6:**
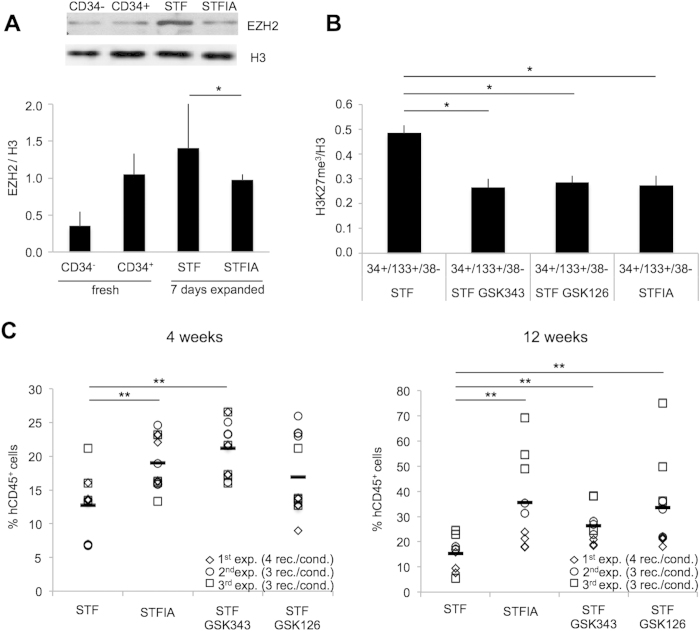
Treatment with PRC2 inhibitors and influence on engrafting activity. **A**) EZH2-specific Western blot analysis of fresh CD34− and CD34+ cells, and of CD34+ cells expanded for 7 days with either STF or STFIA cocktails. Relative signal intensities of bands were quantified (lower panel). Diagram summarizes 3 independent experiments. **B**) CD34+ cells expanded for 7 days with either STF or STFIA cocktails +/− the EZH2 inhibitors GSK343 (1 mM) or GSK126 (1 mM). Intranuclear flow cytometry analysis specific for H3K27me3 and H3 of gated CD34+/CD133+/CD38− cells. Student t-test, *p < 0.05, n = 3. **C**) After 7 days of culture the progeny of 2 × 10^5^ CD34+ cells in STF- or STFIA-supplemented cultures in the absence or presence of either GSK343 or GSK126 EZH2 inhibitors were equally split and injected into recipients. Shown are percentages of human chimerism by human CD45-specific flow cytometry in the peripheral blood of NSG transplant recipients. Each symbol represents the engraftment of a single recipient analyzed 4 and 12 weeks post transplant. All recipients were analyzed and are shown (rec./cond.: recipients per condition). Student t-test, **p < 0.01, n = 3.

Our analyses revealed a culture-associated increase and a genome-wide redistribution of H3K27me3 marks. We therefore assessed the EZH2 protein expression in fresh and expanded CD34+ cells. EZH2 is the protein with the enzymatic activity of PRC2 that catalyzes the H3K27me3 mark^29^. qRT PCR and Western blotting showed higher expression of EZH2 in fresh CD34+ compared to CD34− cells and elevated EZH2 levels in STF- compared to STFIA-cultured cells (Fig. 6A, Fig. S10). ChIPseq analyses of the *EZH2* locus revealed H3K4me3 promoter marking in all samples but higher H3K27me3 levels at the gene body in fresh CD34+ and STFIA- compared to STF-expanded cells (Fig. S10).

Next we hypothesized that employing inhibitors of the histone lysine methyltransferase EZH2 might antagonize the elevated H3K27me3 levels of STF- versus STFIA-cultured cells. Treatment of STF-cultured CD34+ cells with specific EZH2 inhibitors (GSK343 and GSK126^30^,^31^) decreased global H3K27me3 levels comparable to those of CD34+ cells in STFIA culture (Fig. 6B) but did not effect viability or cell numbers during expansion (Fig. S11A). Inhibitor treatment of STF cultures did not raise CFU formation to levels recorded for fresh or STFIA-expanded cultures (Fig. S11B). To assess the impact of inhibitor treatment on *in vivo* hematopoietic engraftment potential we transplanted STF-, STFIA-cultured and STF-plus inhibitor-cultured CD34+ cells into NSG recipients. We observed that treatment of STF-cultured CD34+ cells with the EZH2 inhibitors increased hematopoietic engraftment at 4 and 12 weeks post transplantation to levels of STFIA-cultured CD34+ cells engraftment (Fig. 6C). Recipients of untreated and treated cells showed multilineage hematopoietic engraftment (Fig. S12).

## Discussion

The aim of this study was to provide a better molecular understanding of chromatin changes upon expansion of CB-HPSCs. We observed that globally fresh CD34+ and CD34− cells differed in H3K4me3 and H3K27me3 levels and that expansion remodeled the epigenetic landscape of CD34+ cells. Furthermore, we noticed that global H3K4me3 and H3K27me3 levels changed depending on culture conditions with more changes in H3K27me3- than in H3K4me3-marked regions. Higher protein levels of the PRC2 component EZH2 were detected in STF- compared to STFIA-cultured cells and treatment with EZH2 inhibitors increased hematopoietic engraftment potential of cultured HPSCs.

As previously reported, we confirmed that STFIA-expanded CD34+ cells engrafted NSG recipients^9^. However, we did not assay the self-renewal potential of engrafted CB-HSCs by secondary transplantation. In contrast to an earlier report, engraftment levels of STFIA-cultured cells did not reach the 20-fold net expansion of repopulating HPSCs^9^. We also noticed inter-experimental variations in engraftment similar to the results reported^32^. The variable engraftment could arise from a multitude of factors including how individual HSCs respond to the growth factors and variability in homing.

As the maintenance of HSC function and the differentiation of HSCs are influenced by epigenetic gene regulation^12^, we aimed at dissecting epigenotype changes between fresh HPSCs and HPSCs expanded under different growth factor conditions. Our data of global epigenotypes revealed that H3K4me3 and H3K27me3 levels in CD34+ and CD34− cells differ. Similar to an earlier report we noticed higher global H3K4me3 levels in fresh CD34+ than in fresh CD34− cells^33^.

It needs however to be pointed out that human CB CD34+ cells are a heterogeneous cell population composed of rare quiescent LT HSCs, cycling short-term HSCs and mostly progenitors. Thus, the data collected from this mixed population may overestimate the epigenetic complexity of cell populations with similar potentials and that results with highly purified LT-HSCs would differ. Despite these limitations we observed that the H3K27me3 profiles in the HoxA and HoxB loci of fresh and expanded CD34+ cells were maintained. On average, a single CB unit allows the collection of about 800,000 CD34+ cells. The sample material needed for robust ChIP-seq analyses did not allow the use of higher purified cell subsets. Our isolation strategy is in line with selection strategies of related studies^17^,^18^,^23^. To improve the situation it is important to further optimize ChIPseq profiling for chromatin amounts obtained from a few thousands cells.

Interestingly, culture of HPSCs was paralleled by global changes in H3K27me3 while H3K4me3 levels were less altered. This may indicate that during HPSC expansion PcG-mediated H3K27me3 gene silencing seems functionally more relevant to CD34+ cells than the counteracting trxG-mediated gene activation.

ChIPseq analyses showed that cultured cells lose H3K27me3 marks at the TSSs and over gene bodies and that the size of H3K4me3-enriched regions increased in cultured cells independent of cytokine cocktail. On the other hand, H3K27me3-enriched regions maintained the size distribution of fresh CD34+ cells following STFIA expansion. This is reminiscent with the finding in human HPSCs that H3K4me3 island size distributions are maintained but that H3K27me3 sizes are increased during differentiation^18^. Also consistent with previous observations is the observation of widespread H3K27me3 signals that indicate that H3K27me3 occupied broad domains while H3K4me3 signals are confined to promoter regions^17^,^34^. Our data of variable H3K4me3 and H3K27me3 mark distribution over specific gene regions is consistent with observations that H3K4me3 is found promoter-near while the majority of H3K27me3 marks occur in intergenic regions and forms broad local enrichments (BLOCs) rather than focal peaks^17^,^35^.

Stem and mature cells each have distinct epigenomic landscapes and differentiation is paralleled by large-scale expansion of H3K27me3 but less H3K4me3 domains^34^. The H3K27me3 pattern is highly variable across different cell types and certain regions such as Hox gene clusters are associated with a high-degree of variability^36^. The H3K4me3 and H3K27me3 landscapes seen at the HoxA or B loci are consistent with this notion.

Our analyses further revealed the expected H3K4me3 and H3K27me3 modification profiles around the TSSs of highly and lowly expressed genes^22^. Here we noticed a level of exclusivity of which promoters were H3K4me3- or H3K27me3-marked depending on whether samples from fresh, STF- or STFIA-cultured cells were analyzed. The Venn diagrams in Fig. 4A revealed that one third of the enriched promoters was unique in individual samples. The exclusively marked promoters of fresh and STFIA-expanded CD34+ cells showed similar GO-terms.

Unexpectedly, we observed little transcriptomic changes between STF- and STFIA-cultured cells while changes of H3K27me3 and H3K4me3 levels were apparent upon culture. While further studies are clearly required, one possibility is that the different cocktails primarily act on the epigenomic and less on transcriptomic regulatory systems. Interestingly transcriptome profiling of mouse ESCs and epiblast stem cells, which are naive and primed pluripotent states, reveale small-scale differences in transcriptional output but dramatic alterations in chromatin profiles^37^. This indicates that closely related developmental stages are characterized by altered epigenotypes not yet leading to transcriptomic changes.

The altered epigenotypes also indicate that H3K27me3 and H3K4me3 levels of HPSCs are sensitive to whether the cells are exposed to *in vivo* or *in vitro* environments. This is supported by the observation of a considerable redistribution of bivalent domains upon culture. Although the total number of bivalent promoters was similar in fresh and cultured CD34+ cells, we found approximately two thirds of the genes changing between the different conditions. Thus, although transcriptomic changes were minor between the STF and STFIA culture conditions, different sets of genes were rendered into a transcriptionally permissive state which later may cause differences in engraftment potential. GO-term analysis revealed a cocktail-specific reorganization of the bivalency status. The enrichment of blood vessel development and morphogenesis terms in STFIA cultures may relate to the activity of Angptls^38^ or the angiogenetic signatures may be relevant for HSC expansion.

Finally, the results of this study revealed increased culture-induced EZH2 levels probably regulating its own expression and leading to elevated H3K27 trimethylation levels in STF- compared to STFIA-cultured cells. Therefore, it was obvious to assess if this can be blocked by specific inhibitors. Epigenetic inhibitors have previously been shown to support expansion of CD34+ cells and combination of azaC with trichostatin A supports the maintenance of NSG mice repopulating cells^20^,^39^. The observation that an EZH2 inhibitor treatment counteracted the culture-induced loss of engraftment potential indicates that EZH2 activity may be key to the culture-associated loss of engraftment potential.

Following EZH2 inhibition, we noticed differences *in vivo* but not in *in vitro* hematopoietic activities. This observation may point to an effect of the EZH2 inhibitors on immature HSCs rather than on mature progenitor cells. The increased engraftment levels of EZH2 inhibitor-treated cells were not entirely unexpected as loss of function mutation of the PRC2 core components Suz12, Eed and EZH2 display enhanced hematopoietic progenitor cell activities following transplantation^40^ and overexpression of EZH2 preserves HSCs after replicative stress^41^. Thus, inhibitors of chromatin factors and chromatin-modifying agents provide potential strategies for the expansion of HPSCs. Inhibition of histone methyltransferase activity by small molecule approaches is an active area of research^30^.

In summary, we have provide genome-wide maps for H3K4 and H3K27 trimethylation changes upon expansion of CB-HPSCs and show that the H3K4me3 but less the H3K27me3 landscape is stable. Our data further reveal that inhibition of the PRC2 component EZH2 counteracts the culture-associated loss of engraftment potential. These data may lead to new therapeutic tools and rational protocols for robust expansion of this clinically important adult stem cell type.

## Additional Information

**How to cite this article**: Varagnolo, L. *et al.* PRC2 inhibition counteracts the culture-associated loss of engraftment potential of human cord blood-derived hematopoietic stem and progenitor cells. *Sci. Rep.* **5**, 12319; doi: 10.1038/srep12319 (2015).

## Supplementary Material

Supplementary table 1

Supplementary table 2

Supplementary table 3

Supplementary Information
