# Supplementary material for: PRC2 inhibition counteracts the culture-associated loss of engraftment potential of human cord blood-derived hematopoietic stem and progenitor cells
Source: Sci Rep. 2015 Jul 22;5:12319. doi: 10.1038/srep12319 (PMC4510577; doi:10.1038/srep12319)
Supplement: Supplementary Information [file srep12319-s4.pdf]

Supplementary Informations

PRC2 inhibition counteracts the culture-associated loss of engraftment potential of human cord blood-derived hematopoietic stem and progenitor cells

Linda Varagnolo 1, Qiong Lin 2, Nadine Obier 3, Christoph Plass 4, Johannes Dietl 5, Martin Zenke 2, Rainer Claus 4, 6, Albrecht M. Müller 1

1 Institute of Medical Radiology and Cell Research (MSZ) in the Center for Experimental Molecular Medicine (ZEMM), University of Würzburg, Würzburg, Germany
2 Department of Cell Biology, Helmholtz Institute for Biomedical Engineering, RWTH Aachen University, Aachen, Germany,
3 School of Cancer Sciences, University of Birmingham, Birmingham, United Kingdom,
4 Department of Epigenomics and Cancer Risk Factors, German Cancer Research Center (DKFZ), Heidelberg, Germany,
5 Department of Gynecology and Obstetrics, Medical University of Würzburg, Germany,
6 Department of Medicine, Div. Hematology, Oncology and Stem Cell Transplantation, University of Freiburg Medical Center, Freiburg, Germany

Supplementary figures

Fig. S1

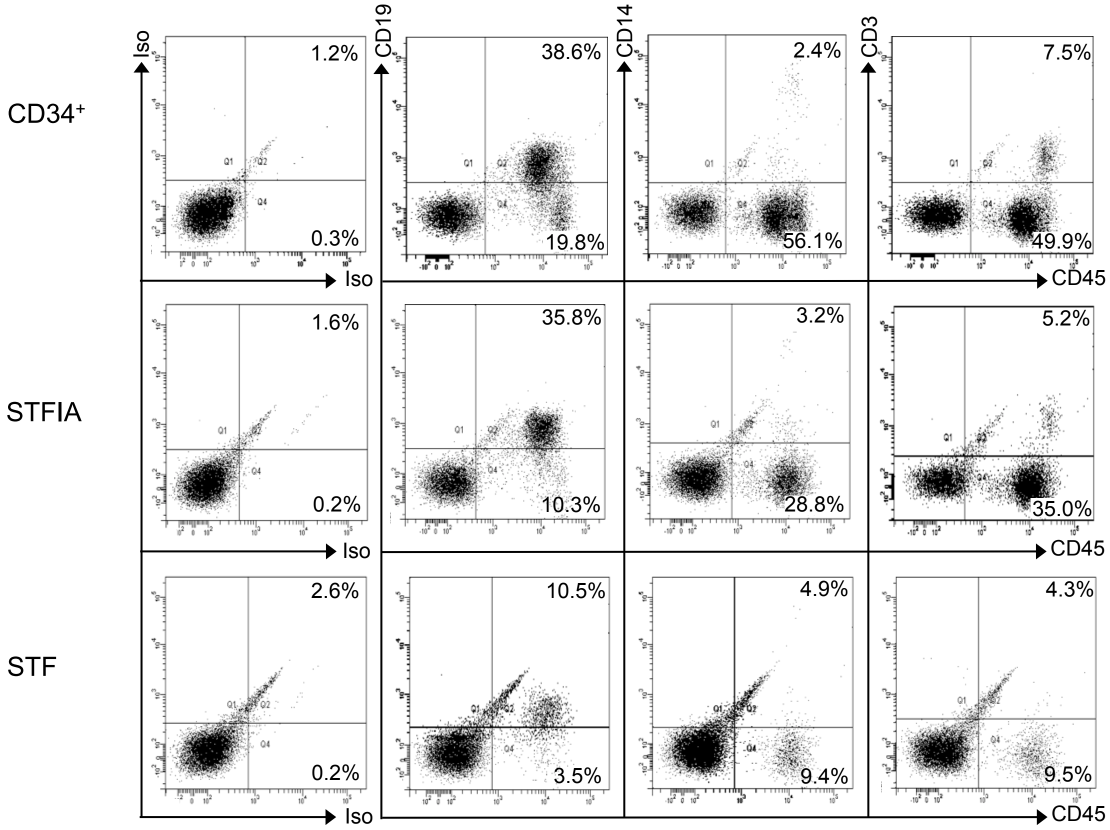

Fig. S2

A

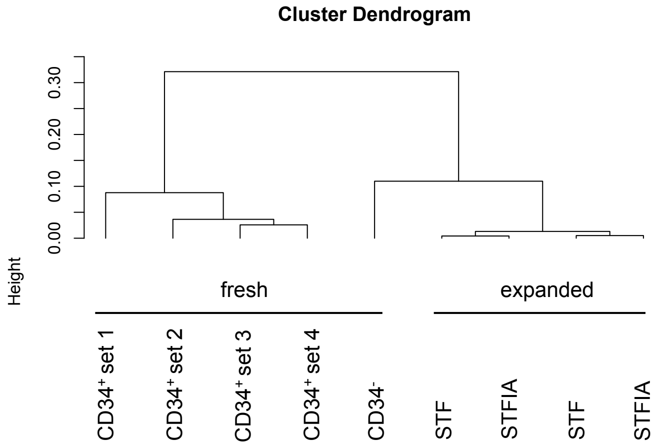
Cluster Dendrogram
Height
0.00
0.10
0.20
0.30
fresh
expanded
CD34+ set 1
CD34+ set 2
CD34+ set 3
CD34+ set 4
CD34-
STF
STFIA
STF
STFIA

B

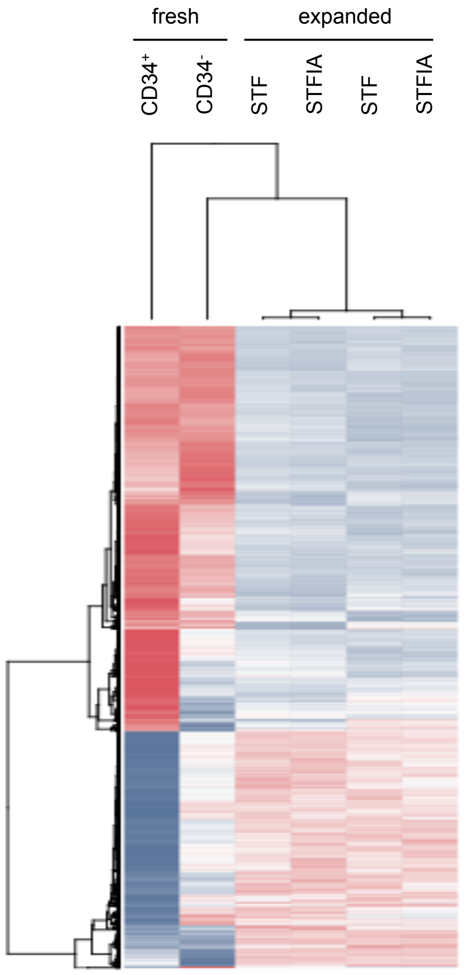
fresh
expanded
CD34+
CD34-
STF
STFIA
STF
STFIA

Fig. S3

Low in STFIA_*versus*_fresh CD34+ cells	Genes count	P-value
regulation of transcription	166	3,2E-2
regulation of RNA metabolic process	65	9,5E-2
regulation of transcription DNA-dependent	64	6,9E-2
regulation of apoptosis	40	1,6E-3
regulation of cell death	80	1,9E-3
immune response	45	1,2E-6
cell death and apoptosis	129	8,0E-3

High in STFIA_*versus*_fresh CD34+ cells	Genes count	P-value
cell cycle process	287	1,5E-43
cell cycle phase and mitotic cell cycle	305	5,1E-39
DNA metabolic process	91	8,6E-20
cell division	81	3,8E-30
nuclear division	75	9,0E-35
mitosis	150	3,5E-34
chromosome organization	69	3,9E-10
macromolecular complex subunit localization	199	3,8E-4

Low in STF_*versus*_fresh CD34+ cells	Genes count	P-value
regulation of transcription	181	1,8E-2
regulation of RNA metabolic process	70	5,7E-2
regulation of transcription DNA-dependent	69	5,2E-2
regulation of apoptosis	37	2,2E-2
regulation of cell death	74	2,5E-2
immune response	45	7,2E-6
cell death and apoptosis	93	3,4E-2

High in STF_*versus*_fresh CD34+ cells	Genes count	P-value
cell cycle process	309	2,5E-40
cell cycle phase and mitotic cell cycle	325	1,6E-35
DNA metabolic process	103	1,3E-20
cell division	85	1,3E-27
nuclear division	79	7,3E-33
mitosis	158	6,7E-33
chromosome organization	79	2,0E-9
macromolecular complex subunit localization	161	1,3E-4

Fig. S4

A

H3K4me3

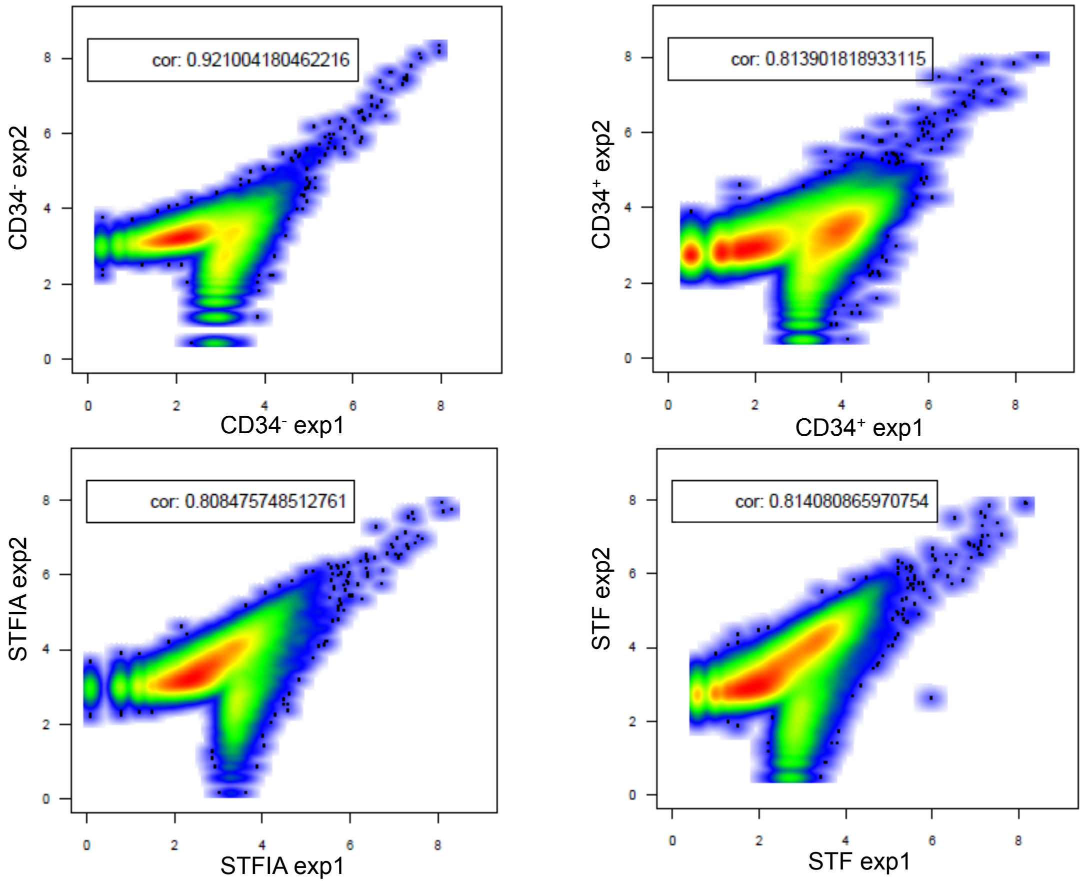

B

H3K27me3

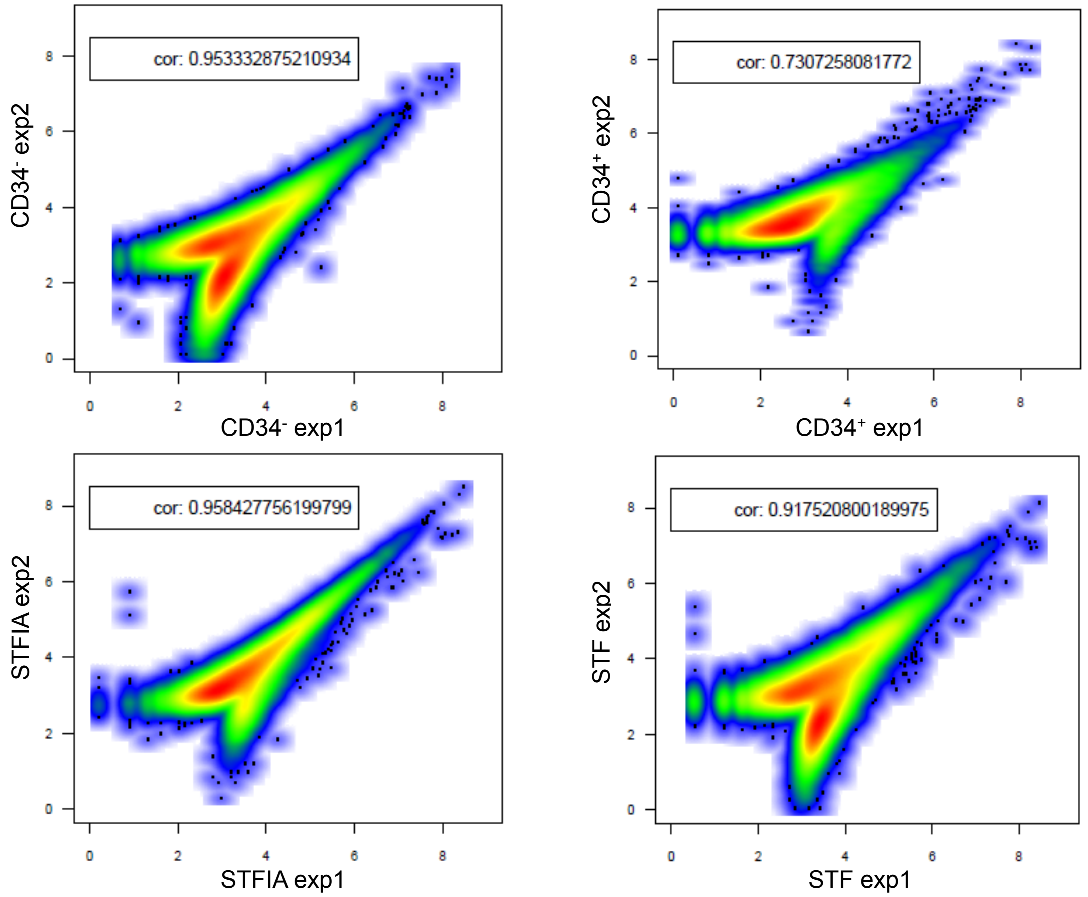

Fig. S5

A

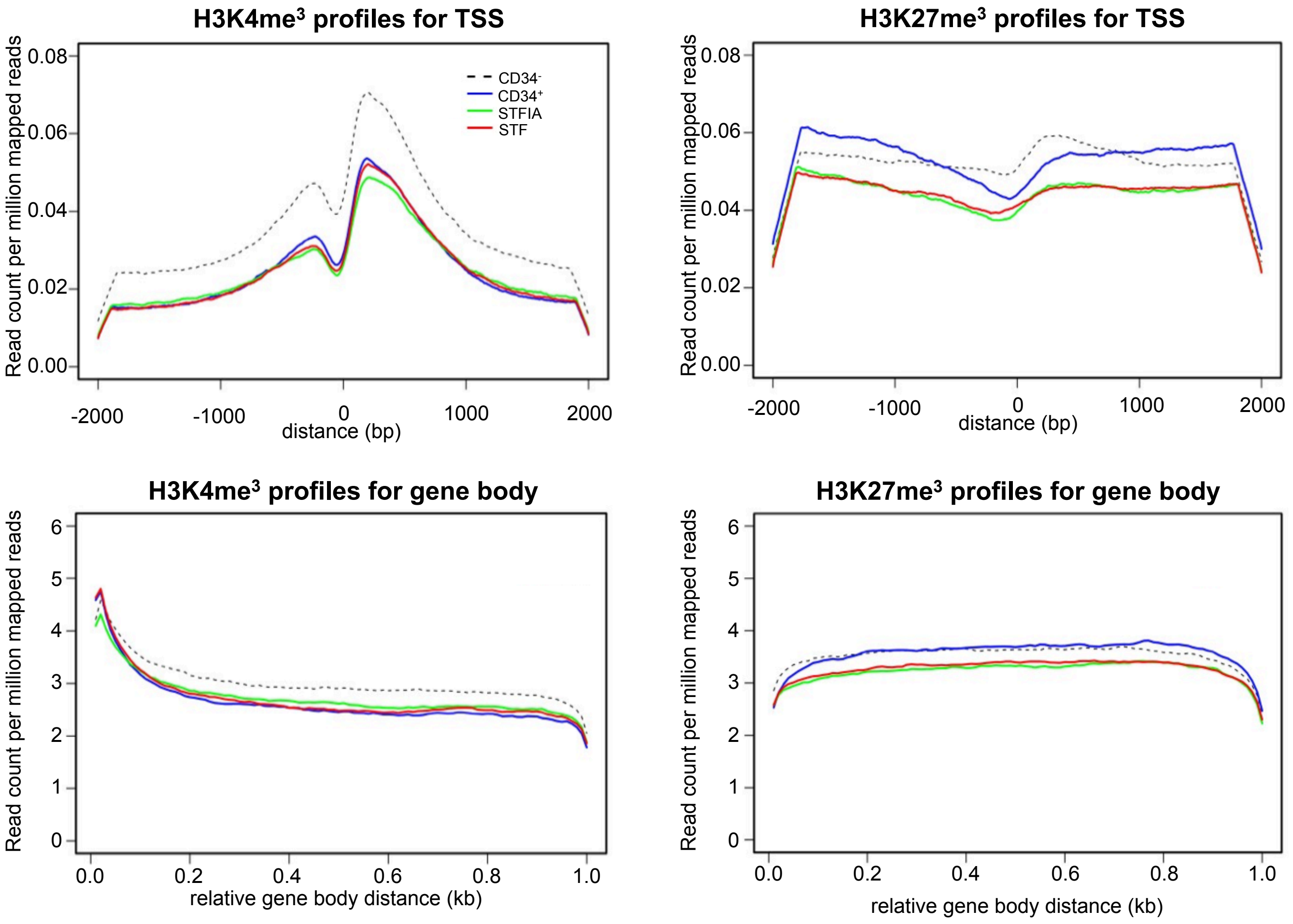
H3K4me3 profiles for TSS
H3K27me3 profiles for TSS
H3K4me3 profiles for gene body
H3K27me3 profiles for gene body
Read count per million mapped reads
distance (bp)
relative gene body distance (kb)
CD34-
CD34+
STFIA
STF

B

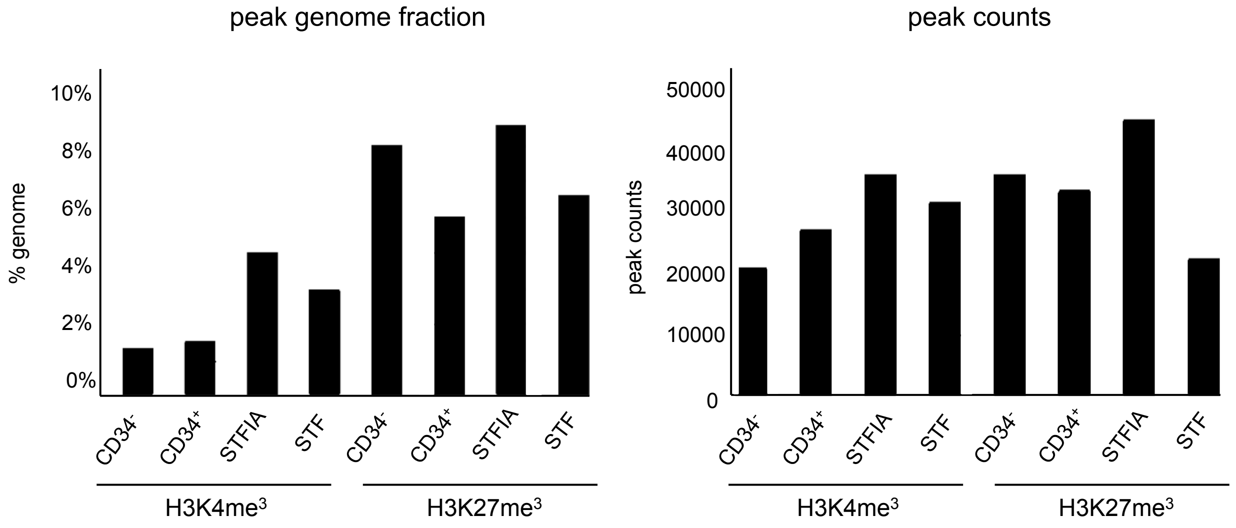
peak genome fraction
peak counts
% genome
peak counts
CD34-
CD34+
STFIA
STF
H3K4me3
H3K27me3

Fig. S6

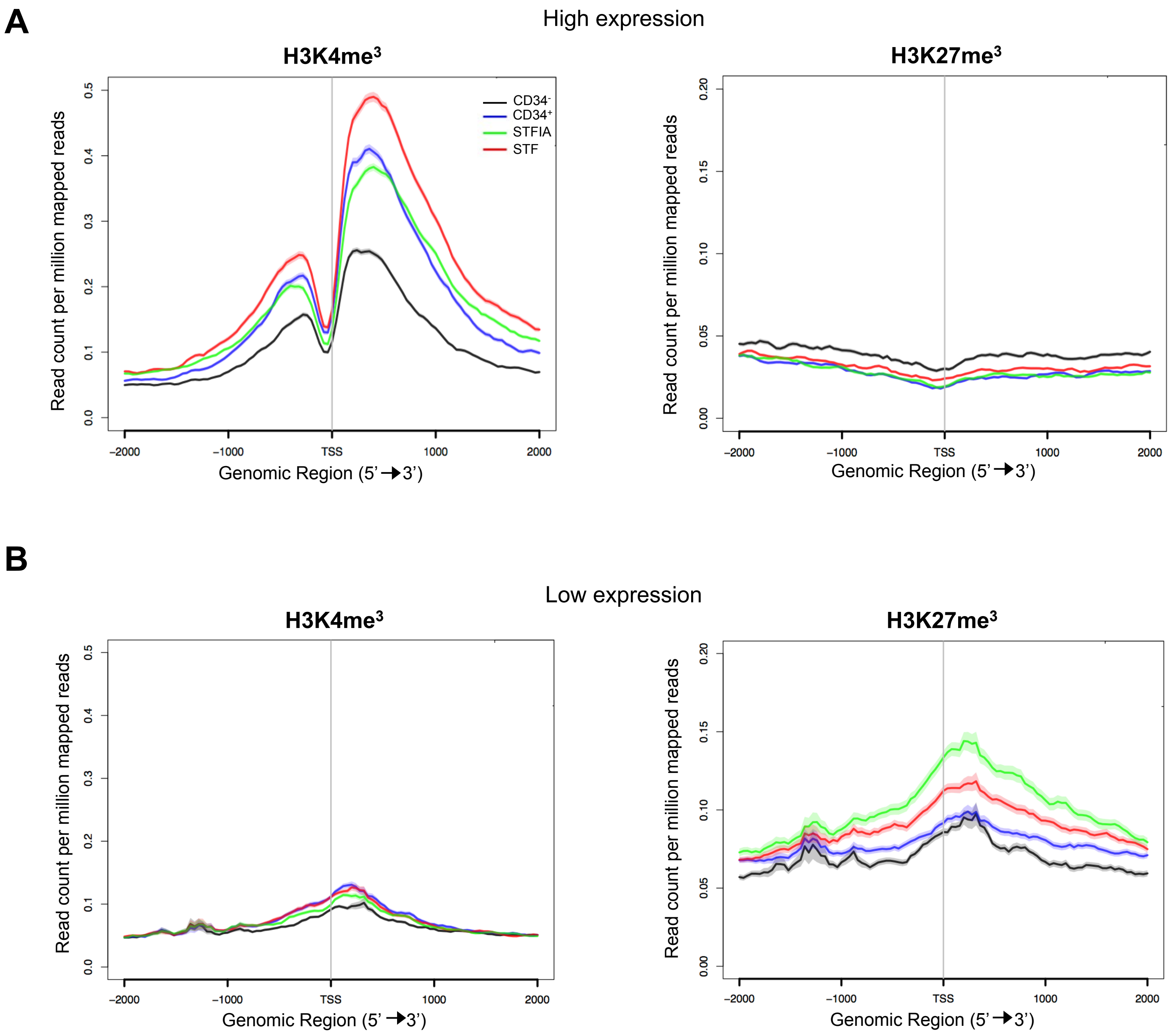
A
High expression
H3K4me3
CD34-
CD34+
STFIA
STF
Read count per million mapped reads
Genomic Region (5' → 3')
−2000
−1000
TSS
1000
2000
H3K27me3
B
Low expression
H3K4me3
H3K27me3

Fig. S7

A

H3K4me^3

CD34+	Genes count/ 1772	P-value
regulation of transcription	247	8.4E-3
transcription	204	7.3E-3
regulation of transcription DNA-dependent	164	7.5E-2
cell cycle	92	3.7E-4
positive regulation of macromolecule metabolic process	90	1.4E-2
protein localization	90	2.8E-2
macromolecular complex subunit localization	83	1.1E-3
establishment of protein localization	83	9.8E-3
protein transport	81	1.5E-2
macromolecular complex assembly	78	1.5E-3
regulation of transcription from RNA polymerase II promoter	77	1.9E-2
negative regulation of macromolecule metabolic process	76	3.3E-2
intracellular transport	75	3.8E-3

STFIA	Genes count/ 1436	P-value
regulation of transcription	187	6.4E-2
regulation of RNA metabolic process	135	4.9E-2
regulation of transcription DNA-dependent	131	6.3E-2
intracellular signaling cascade	94	8.6E-2
phosphate metabolic process	82	1.0E-2
phosphorus metabolic process	82	1.0E-2
positive regulation of macromolecule metabolic process	71	2.4E-2
response to organic substances	63	1.2E-2
regulation of cell proliferation	63	5.8E-2
intracellular transport	61	4.0E-3
regulation of transcription from RNA polymerase II promoter	61	2.9E-2
cellular macromolecular catabolic process	58	6.9E-2
positive regulation of hydrogen compound metabolic process	55	2.8E-2

STF	Genes count/ 1407	P-value
intracellular signaling cascade	100	2.3E-2
phosphorus metabolic process	86	2.9E-3
phosphate metabolic process	86	2.9E-3
regulation of cell death	73	4.6E-3
regulation of programmed cell death	71	9.1E-3
phosphorylation	70	1.0E-2
regulation of apoptosis	70	1.1E-2
cell cycle	68	4.4E-2
macromolecular catabolic process	64	4.1E-2
cellular macromolecular catabolic process	60	6.9E-2
cell death	58	7.6E-2
death	58	2.9E-2
protein amino acid phosphorylation	57	9.4E-2

H3K27me^3

CD34+	Genes count/ 513	P-value
cell surface receptor linked signal transduction	67	3.4E-4
regulation of transcription DNA-dependent	52	7.5E-2
neurological system process	50	1.2E-4
intracellular signaling cascade	45	5.0E-4
G-protein coupled receptor protein signaling pathway	41	2.0E-6
cell-cell signaling	35	9.0E-4
response to organic substance	32	2.4E-3
ion transport	32	2.4E-2
cognition	32	1.7E-6
neuron differentiation	29	7.3E-3
regulation of transcription from RNA polymerase II promoter	29	2.0E-2
regulation of cell proliferation	29	2.2E-5
cell motion	28	6.3E-3

STFIA	Genes count/ 679	P-value
cell surface receptor linked signal transduction	75	5.9E-3
G-protein coupled receptor protein signaling pathway	43	8.1E-2
regulation of transcription DNA-dependent	41	4.7E-4
cell adhesion	39	3.0E-4
biological adhesion	39	3.0E-4
regulation of cell proliferation	34	3.3E-2
cell-cell signaling	30	8.0E-3
neuron differentiation	29	1.4E-4
defense response	29	1.9E-2
cellular component morphogenesis	28	6.6E-5
cell motion	28	1.1E-3
cation transport	28	9.0E-3
neuron development	26	3.3E-5

STF	Genes count/ 395	P-value
cell surface receptor linked signal transduction	57	1.4E-4
neurological system process	36	5.9E-3
intracellular signaling cascade	33	3.8E-1
G-protein coupled receptor protein signaling pathway	32	3.0E-2
cell-cell signaling	27	1.5E-1
ion transport	27	2.4E-1
protein amino acid phosphorylation	23	5.4E-1
phosphorylation	23	1.4E-1
cation transport	22	4.8E-1
regulation of transcription from RNA polymerase II promoter	22	1.5E-2
transmission of nerve impulse	21	8.0E-2
metal ion transport	21	4.9E-1
positive regulation of cellular biosynthetic process	21	5.2E-1

Fig. S8

A

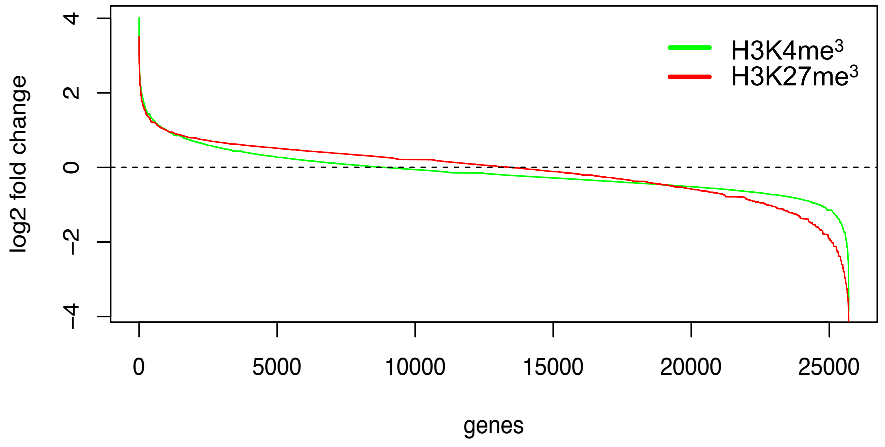

log2 fold change
4
2
0
−2
−4
H3K4me3
H3K27me3
0
5000
10000
15000
20000
25000
genes

B

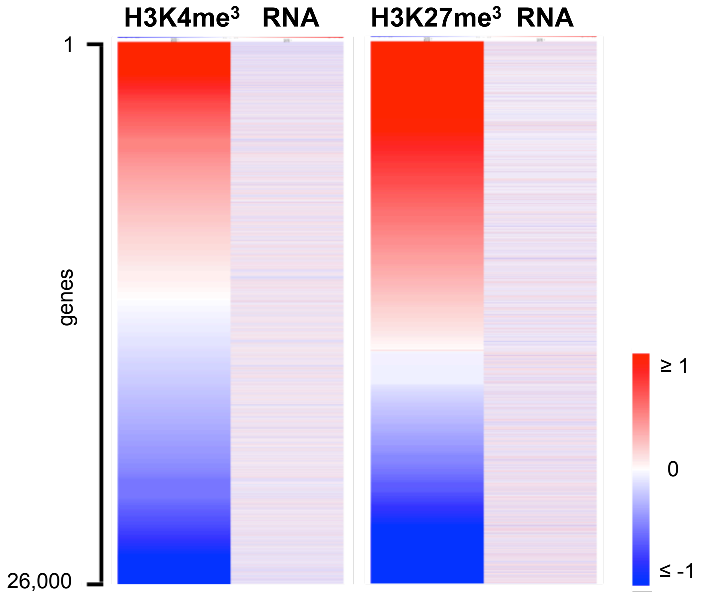

H3K4me3
RNA
H3K27me3
RNA
1
genes
26,000
≥ 1
0
≤ -1

C

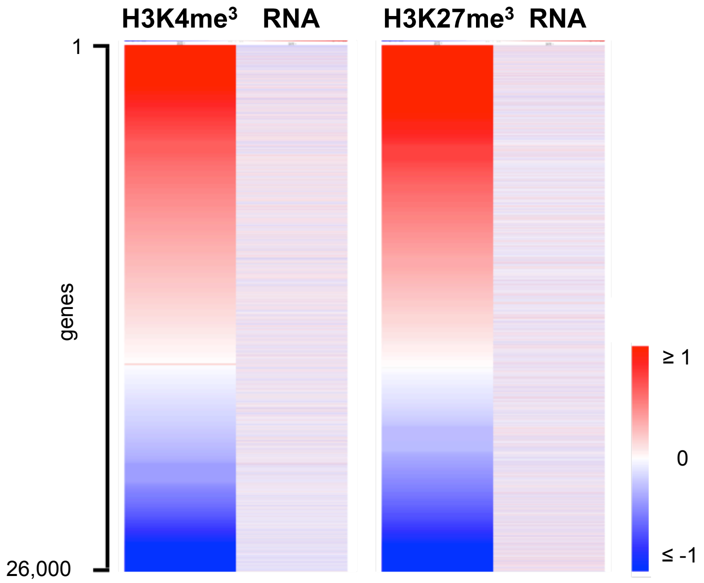

H3K4me3
RNA
H3K27me3
RNA
1
genes
26,000
≥ 1
0
≤ -1

Fig. S9

CD34+	P-value
cell-cell signaling	3.4E-9
developmental induction	2.3E-5
cell-cell signaling involved in cell fate specification	2.3E-5
gland development	5.5E-5
ion transport	6.3E-5
induction of an organ	8.1E-5
behavior	9.6E-5
neuron differentiation	2.8E-4
tube development	3.1E-4
cell fate commitment	3.3E-4
inorganic anion transport	4.0E-4
positive regulation of locomotion	5.6E-4

STFIA	P-value
blood vessel development	9.5E-6
vasculature development	1.3E-5
cell-cell signaling	1.9E-4
regulation of cell development	2.6E-4
blood vessel morphogenesis	3.5E-4
muscle organ development	3.5E-4
cell morphogenesis involved in differentiation	4.3E-4
regulation of striated muscle tissue development	4.7E-4
regulation of muscle development	5.3E-4
axogenesis	5.4E-4
response to alkaloid	7.2E-4
response to hormone stimulus	1.1E-3

STF	P-value
cell-cell signaling	1.2E-6
regulation of axogenesis	5.0E-6
metal ion transport	1.5E-5
ion transport	1.7E-6
potassium ion transport	1.8E-5
regulation of cell morphogenesis involved in differentiation	4.7E-5
regulation of cell morphogenesis	7.9E-5
regulation of neuron differentiation	9.0E-5
regulation of cell projection organization	1.3E-4
cation transport	1.9E-4
cell surface receptor linked signal transduction	5.4E-4
regulation of cell development	7.7E-4

CD34-	P-value
neuron differentiation	2.0E-4
axon guidance	4.7E-4
cell projection organization	6.0E-4
cellular component morphogenesis	1.1E-3
regulation of neuron differentiation	1.5E-3
gland development	1.6E-3
cell morphogenesis involved in differentiation	1.9E-3
axogenesis	2.1E-3
neuron projection development	2.6E-3
regulation of cell development	2.9E-3
cell morphogenesis involved in neuron differentiation	3.3E-3
neuron projection morphogenesis	3.6E-3

Fig. S10

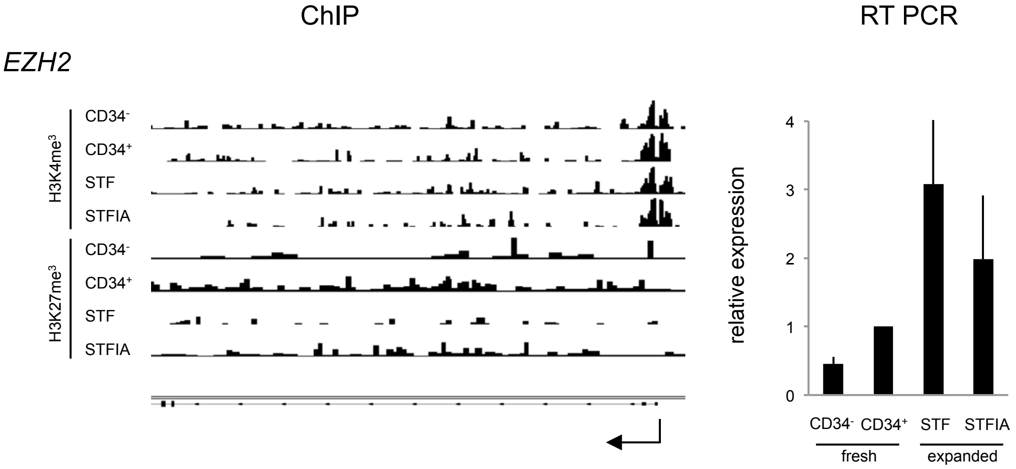
ChIP
RT PCR
EZH2
H3K4me3
H3K27me3
CD34-
CD34+
STF
STFIA
relative expression
0
1
2
3
4
CD34-
CD34+
STF
STFIA
fresh
expanded

Fig. S11

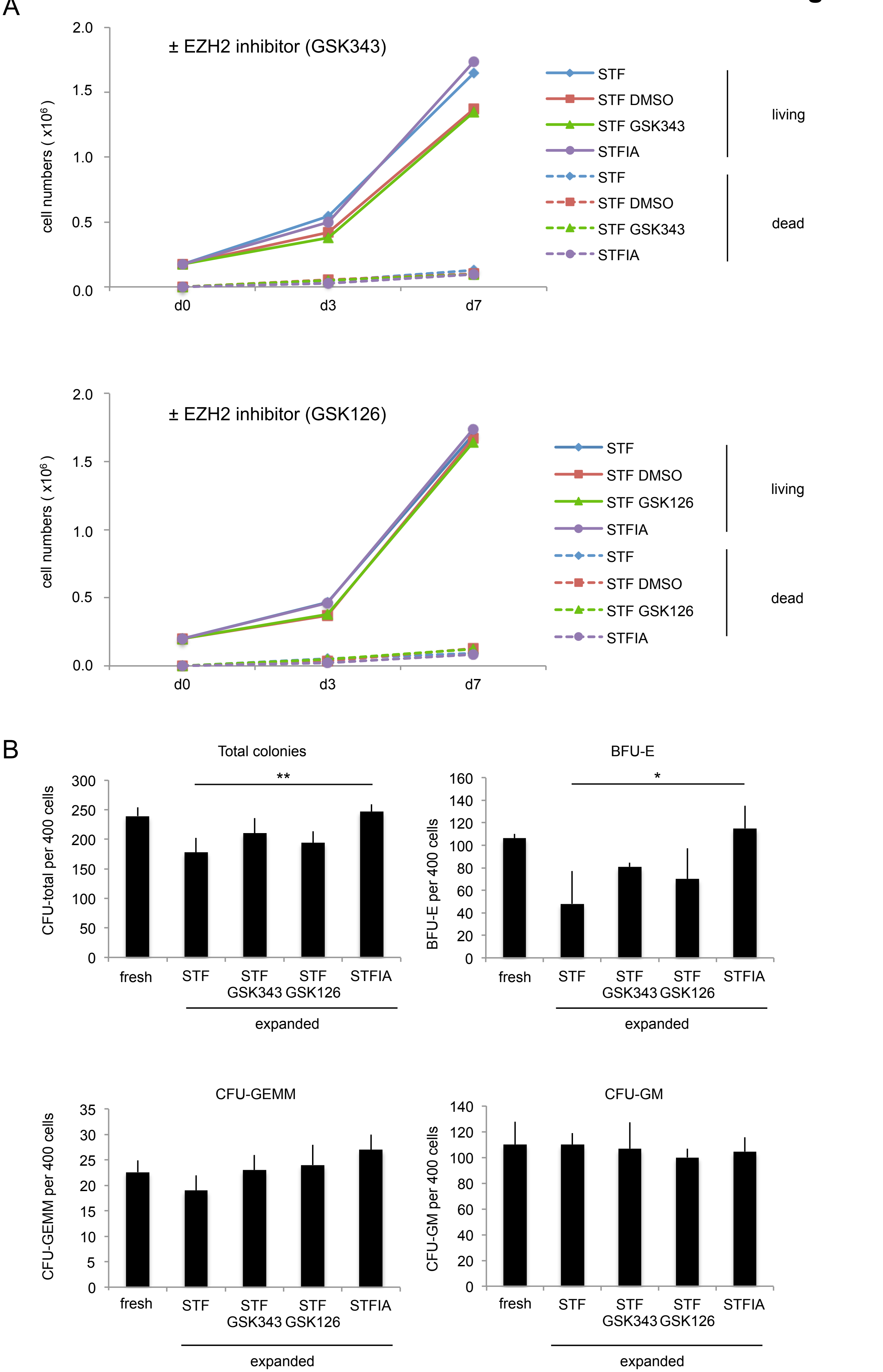
A
± EZH2 inhibitor (GSK343)
cell numbers (x10⁶)
2.0
1.5
1.0
0.5
0.0
d0
d3
d7
STF
STF DMSO
STF GSK343
STFIA
living
STF
STF DMSO
STF GSK343
STFIA
dead
± EZH2 inhibitor (GSK126)
cell numbers (x10⁶)
STF
STF DMSO
STF GSK126
STFIA
living
STF
STF DMSO
STF GSK126
STFIA
dead
B
Total colonies
**
CFU-total per 400 cells
300
250
200
150
100
50
0
fresh
STF
STF GSK343
STF GSK126
STFIA
expanded
BFU-E
*
BFU-E per 400 cells
160
140
120
100
80
60
40
20
0
CFU-GEMM
CFU-GEMM per 400 cells
35
30
25
20
15
10
5
CFU-GM
CFU-GM per 400 cells

Fig. S12

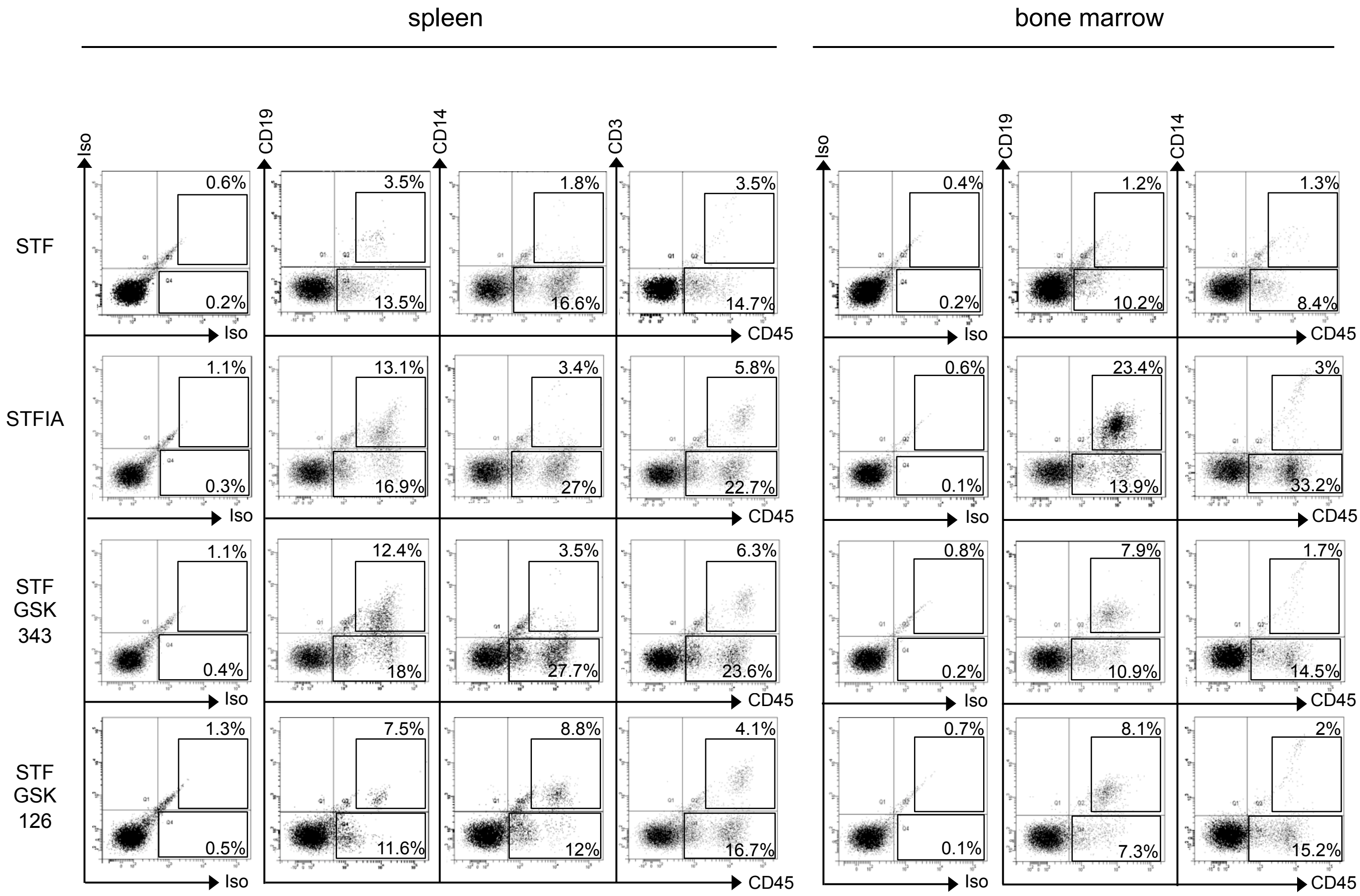
spleen
bone marrow
STF
STFIA
STF GSK 343
STF GSK 126
Iso
CD19
CD14
CD3
CD45
0.6%
0.2%
3.5%
13.5%
1.8%
16.6%
3.5%
14.7%
0.4%
0.2%
1.2%
10.2%
1.3%
8.4%
1.1%
0.3%
13.1%
16.9%
3.4%
27%
5.8%
22.7%
0.6%
0.1%
23.4%
13.9%
3%
33.2%
1.1%
0.4%
12.4%
18%
3.5%
27.7%
6.3%
23.6%
0.8%
0.2%
7.9%
10.9%
1.7%
14.5%
1.3%
0.5%
7.5%
11.6%
8.8%
12%
4.1%
16.7%
0.7%
0.1%
8.1%
7.3%
2%
15.2%

Supplementary figure legends

Figure S1: Multilineage engraftment analyses in NSG recipients.

Representative analysis of human chimerism in the spleen of transplant recipients of fresh CD34+ or STF- or STFIA-expanded CD34+ cells. Splenocytes of animals were analyzed via flow cytometry 8 weeks post transplantation using antibodies specific for human hematopoietic cells. Percentages of positive cells are indicated.

Figure S2: Hierarchical clustering and heat maps of global gene expression profiles of fresh CD34- and CD34+ and expanded CD34+ cells.

A) Hierarchical cluster dendrogram of whole gene expression datasets indicating the relatedness in total gene expression. For fresh CD34+ cells, 4 published datasets were used (GSM999015, GSM999018, GSM999021, GSM1139830). All datasets, comprising the published datasets, were generating using the Affymetrix Human Gene 1.0 ST Array platform. B) Heatmaps of differentially expressed genes in fresh and expanded cells. In this representation, samples that share similar expression profiles have closer Euclidean distances to common branch points and are grouped. Gene expression levels are color-coded (blue, low expression; red, high expression).

Figure S3: Gene ontology enrichment analysis of differentially expressed genes between fresh CD34+ cells and CD34+ cells expanded in STF and STFIA.

Functional annotation analysis of higher and lower expressed genes in the fresh $CD34^{+}$ sample compared to STF and STFIA samples. Gene ontology analyses were performed with Database for Annotation, Visualization and Integrated Discovery (DAVID). Gene counts and p-values are indicated.

Figure S4: ChIPseq raw data processing and quality control.

Two independent ChIPseq experiments per biological condition were compared before merging. Correlations of sequencing tag counts between replicates in peak regions were plotted (using logarithmic scale) and calculated using Pearson's correlation coefficient. The correlation coefficients are indicated. Replicates were pooled according to ENCODE ChIPseq guidelines, consecutively. Finally, reads were normalized to 10 millions in all samples.

Figure S5: ChIPseq analyses on promoter and gene body regions and peak genome fraction of H3K4me3- and H3K27me3-marked regions.

A) H3K4 and H3K27 trimethylation profiles at promoter and gene body regions in fresh CD34-, CD34+, and in 7 days STF- or STFIA-expanded CD34+ cells. Y axes display read density per base pair. Gene body distances are displayed as percentage of variable gene body size. B) Shown are H3K4me3 and H3K27me3 absolute peak count numbers (after peak calling using SICER) in the different datasets (right panel). The percentages of genome covered by significantly enriched regions (peaks) is displayed for H3K4me3 and H3K27me3 histone marks for fresh and expanded cells (left panel).

Figure S6: Correlation of H3K4me3 and H3K27me3 marks and gene expressions.

A) H3K4me3 and H3K27me3 profiles around the TSSs of the 2000 highest expressed genes in fresh CD34+ cells of fresh CD34- and CD34+ cells, and of CD34+ cells expanded for 7 days with either STF or STFIA cocktails. B) H3K4me3 and H3K27me3 profiles around the TSSs of the 2000 lowest expressed genes in fresh CD34+ cells.

Figure S7: Gene ontology analyses of H3K4me3- and H3K27me3-enriched promoters.

Functional annotation analysis of enriched promoters in fresh and expanded CD34+ cells using gene ontology analysis with Database for Annotation, Visualization and Integrated Discovery (DAVID). Gene counts and p-values are indicated. Color coding as in Fig. 4A.

Figure S8: Correlation between histone modification changes on promoters and gene expression.

A) Fold changes of H3K27me3 and H3K4me3 between STFIA- and STF-cultured samples were sorted by amplitude and plotted as line plots. The area below the curve indicates the relative extent of changes of either histone modification. B, C) Approximately 26,000 transcripts taken from Affymetrix expression arrays of fresh CD34+ cells and of STFIA- or STF-cultured CD34+ cells were ranked according to log2-fold changes of sequencing reads. Shown are B) STFIA *versus* CD34+ and C) STF *versus* CD34+ promoter regions (defined as -1000 bp to +500 bp around transcriptional start sites).

Log2-fold changes (STFIA *versus* CD34+ (H3K4me3 ranged from (-3.83) to (4.51); H3K27me3 from (-4.45) to (4.81) ; STF *versus* CD34+ (H3K4me3 ranged from (-3.83) to (4.19); H3K27me3 from (-4.32) to (4.55)) were displayed by color coding (shades of red = positive log2-fold changes, grey = no change/0, blue = negative log2-fold changes). The same representation was chosen for the log2-fold changed mRNA expression for the respective transcripts (lanes labeled 'RNA').

Figure S9: Gene ontology analyses of bivalent promoters.
Functional annotation analysis of bivalent promoters in fresh and expanded CD34+ cells using gene ontology analysis with Database for Annotation, Visualization and Integrated Discovery (DAVID). p-values are indicated.

Figure S10: H3K4me3, H3K27me3 modification and gene expression in the CD34 and EZH2 loci.
ChIPseq profiles and RTPCR analyses of EZH2 (chr7:148,502,464-148,583,441) loci with fresh CD34- and CD34+ cells and with 7 days STF- and STFIA-expanded cells.

Figure S11: Cell counts, viability and hematopoietic colony formation of EZH2 inhibitor-treated CD34+ cells.
A) $0.2x10^6$ CD34+ cells were expanded for 7 days with either STF or STFIA cocktails +/- the EZH2 inhibitors GSK343 (1 mM) or GSK126 (1 mM) and total living and dead cells were counted (Trypan blue staining) at the indicated time points, n=3. B) After 7 days of expansion with either STF or STFIA cocktails +/- the EZH2 inhibitors, 400 cells were harvested and seeded into methylcellulose cultures supplied with hematopoietic growth factors to evaluate the clonogenic potential. Total colony numbers and BFU-E, CFU-GEMM and CFU-GM frequencies per 400 cells are shown. Results express mean ± SD of two independent experiments performed in triplicate. Student t-test, * $p< 0.05$, ** $p<0.01$.

Figure S12: Multilineage engraftment analyses in NSG recipients.
Representative analysis of human chimerism in the spleen and bone marrow of transplant recipients of STF- or STFIA-expanded CD34+ cells, or of STF-expanded CD34+ cells treated with either GSK343 or GSK126. Animals were analyzed via flow cytometry 8 weeks post transplantation using antibodies specific for human hematopoietic cells. Percentages of positive cells (boxed in the figure) are indicated.

RNA preparation, qRT PCR and global gene expression analysis

RNA isolation was performed using the RNeasy Micro kit (Qiagen). For qRT-PCR analyses ABsolute SybrGreen Mix (ThermoFisher) and gene-specific primers were used. The samples were run on a RG-3000 (Corbett). Expression level of individual genes was referred to ß-actin and RPL27 expression. Primers used are listed in Table S5. For microarray analysis RNAs were isolated using RNeasy Mini Kit und DNase I digestion (Qiagen). RNA quality was analyzed using the Agilent RNA 6000 Pico Total RNA Kit with a 2100 Bioanalyzer (Agilent Technologies Genomics). Briefly, sample preparation was performed according to the Expression Analysis Technical Manual (Affymetrix), and all arrays were generated in the microarray faculty of the RWTH Aachen. GeneChip One-cycle Target Labeling Kit (Affymetrix) and 1 µg total RNAs were used. Biotin-labeled cRNAs were hybridized onto Affymetrix Human Gene 1.0 ST arrays. For minimizing batch effects all arrays including the published datasets used the Affymetrix Human Gene 1.0 ST Array platform. Arrays were stained, washed, and scanned according to the manufacturer's protocols. Gene expression levels were determined by RMA algorithm using Affymetrix power tools. Hierarchical clustering was performed using Pearson correlation coefficient and the average linkage method and represented by dendrogram and heatmap. The transcripts having a >2-fold change were considered as being differentially expressed. Data sets were submitted to Gene Expression Omnibus database (www.ncbi.nlm.nih.gov/geo) under accession number GSE58461.

Chromatin immunprecipitation (ChIP)

ChIP was done with adjustments to a protocol described by [1]. Chromatin was sheared with a Covaris M220 focused-ultrasonicator (15 min, 10% duty factor). The DNA fragment size and quantification was analyzed using the Agilent High Sensitivity DNA kit with a 2100 Bioanalyzer (Agilent Technologies Genomics). 10% of the chromatin was used as input material. 2.5 µg of H3K4me3 (Abcam), H3K27me3 (Diagenode), H3 (Abcam) or 1 µg of IgG isotype control (Abcam) antibodies were used per 100ul chromatin solution. DNAs were dissolved in 40 µl TE buffer prior use for qPCR. 2 µl of ChIP-DNA and input DNA were used per PCR reaction together with ABsolute SybrGreen Mix (ThermoFisher) and ChIP-specific primers. Analyses were referred to non-precipitated input DNA. Values were normalized for H3 ChIP-DNA levels. The primers used were specific for the promoter regions (Table S6).

ChIPseq analysis

DNA from ChIP (10-30 ng) was used for library preparation (Illumina). Adapter ligated and amplified fragments were sequenced on an Illumina HiSeq 2000 sequencing system using 50 bp single end reads. Sequence tags were mapped to the human reference sequence version GRCh37/hg19 using BWA [2]. Reads were filtered for unique mapping and mapping quality. Duplicate reads were removed. Supplemental Table S4 summarizes the ChIP-seq data (uniquely mapped, deduplicated reads). Sequence tags from biological replicates were analyzed for correlation/reproducibility using ENCODE criteria [3]. Peaks were called separately in replicate samples, peak areas of replicates were then merged, and sequence tags in merged peak regions were finally correlated between replicates. The distribution of log-transformed ChIP-seq tags in replicates was plotted as a color-coded tag count density map. Tags from replicates were combined and normalized to 10^7 tags per sample. The fraction of reads falling within peak regions (fraction of reads in peaks, FRiP) was used as a measure for global ChIP enrichment. For global similarity analyses, tags were counted into 500 bp genomic bins and unsupervised hierarchical clustering was applied using Ward's minimum variance method. As H3K4me3 and H3K27me3 were expected to cover larger sized regions of the genome, SICER version 1.1 [4] was used for peak detection with a fragment size estimate of 150 bp, a window size of 200 bp and a gap size of 400 bp for H3K4me3 and 600 bp for H3K27me3. FDR cut-off for statistical enrichment was set to 1×10^{-2}. Genomic locations of peaks were defined relative to RefSeq transcription start sites (TSSs) and annotated using HOMER [5]. Promoters were defined from -1kb to +100bp. Promoters were considered to be bivalent if they had significant enrichment for both H3K4me3 and H3K27me3 within the narrow promoter window. The enrichment of Gene Ontology terms was calculated using DAVID tools [6] and sorted by term enrichment p-value. Histograms of tag densities were calculated with position-corrected, normalized tag counts using the ngs.plot software [7]**.** We used MeV [8] to generate heatmaps, and area-proportional Venn diagrams were created (VennDiagram package in R, http://cran.r-project.org/web/packages/VennDiagram). UCSC browser tracks were created with a resolution of 1 bp window and normalized to 10^7 reads using HOMER. For relating changes of chromatin modifications and mRNA expression data, app. 26,000 transcripts with unique genomic representation and processable mRNA expression data read out on the Affymetrix arrays were chosen and respective promoter regions were retrieved using the BiomaRt package [9]. Genomic intervals 1,000 bp upstream and 500 bp downstream the transcriptional start sites of those transcripts were defined and sequence tags from normalized ChiP-seq data were counted into the intervals. Log2-fold changes were calculated for ChiP-seq reads and for Affymetrix based expression data. Heatmaps were created using the Multiple Experiment Viewer (MeV, http://www.tm4.org).

Colony-forming unit (CFU) assays

For analysis of clonogenicity 35-mm Petri dishes containing serum-free medium (MethoCult SF H4236) were used. 400 cells originating from day 7 STF-supplemented CD34+ expansion cultures +/- EZH treatment were seeded into methylcellulose supplemented with growth factors listed in Table S7 (three triplicates per condition), and incubated in a humidified atmosphere at 37°C and 5% CO_2 for 14 days. In addition, 400 STFIA-expanded CD34+ cells were seeded into methylcellulose cultures. 400 freshly isolated CD34+ cells were seeded and scored as controls. After 14 days of incubation, numbers of granuloid/myeloid colonies (colony-forming unit granulocyte-macrophage (CFU-GM)), multilineage colonies (colony-forming unit granulocyte-erythrocyte-macrophage-megakaryocyte (CFU-GEMM)) and erythroid colonies (burst forming unit-erythrocyte (BFU-E)) were scored using an inverted light microscope.

Table S4: Summary of ChIP seq data

Cell type/culture condition	Epitope	Replicate	Total uniquely mapped, deduplicated reads
**CD34- (d0)**	H3K27me3	1	9029235
**CD34- (d0)**	H3K27me3	2	15663007
**CD34- (d0)**	H3K27me3 (input)	1	24725732
**CD34- (d0)**	H3K27me3 (input)	2	28589353
**CD34- (d0)**	H3K4me3	1	14754650
**CD34- (d0)**	H3K4me3	2	6443809
**CD34- (d0)**	H3K4me3 (input)	1	16096097
**CD34- (d0)**	H3K4me3 (input)	2	33016401
**CD34+ (d0)**	H3K27me3	1	10179618
**CD34+ (d0)**	H3K27me3	2	26803042
**CD34+ (d0)**	H3K27me3 (input)	1	23307167
**CD34+ (d0)**	H3K27me3 (input)	2	39967420
**CD34+ (d0)**	H3K4me3	1	5852787
**CD34+ (d0)**	H3K4me3	2	12151887
**CD34+ (d0)**	H3K4me3 (input)	1	7211618
**CD34+ (d0)**	H3K4me3 (input)	2	29163804
**CD34+ STF (d7)**	H3K27me3	1	5859877
**CD34+ STF (d7)**	H3K27me3	2	38385914
**CD34+ STF (d7)**	H3K27me3 (input)	1	26100808
**CD34+ STF (d7)**	H3K27me3 (input)	2	37173902
**CD34+ STF (d7)**	H3K4me3	1	11123244
**CD34+ STF (d7)**	H3K4me3	2	12207286
**CD34+ STF (d7)**	H3K4me3 (input)	1	10699618
**CD34+ STF (d7)**	H3K4me3 (input)	2	27582144
**CD34+ STFIA (d7)**	H3K27me3	1	8057867
**CD34+ STFIA (d7)**	H3K27me3	2	30173754
**CD34+ STFIA (d7)**	H3K27me3 (input)	1	24429976
**CD34+ STFIA (d7)**	H3K27me3 (input)	2	44779700
**CD34+ STFIA (d7)**	H3K4me3	1	9214270
**CD34+ STFIA (d7)**	H3K4me3	2	16611645
**CD34+ STFIA (d7)**	H3K4me3 (input)	1	9156193
**CD34+ STFIA (d7)**	H3K4me3 (input)	2	28961539

Table S5: List of RT PCR primers (5' – 3')

Gene	Sequence forward primer	Sequence reverse primer
EZH2	AGGAGTTTGCTGCTGCTCTC	CCGAGAATTTGCTTCAGAGG
HOXA6	AAAGCACTCCATGACGAAGGCG	TCCTTCTCCAGCTCCAGTGTCT
HOXA9	AGAATGAGAGCGGCGGAGACAA	CTCTTTCTCCAGTTCCAGGGTC
HOXB4	ACACCCGCTAACAAATGAGG	GCACGAAAGATGAGGGAGAG
RPL27	ATCGCCAAGAGATCAAAGATAA	TCTGAAGACATCCTTATTGACG
BETA-ACTIN	GCTATCCCTGTACGCCTCTG	CTCCTTCTGCATCCTGTCGG

Table S6: List of ChIP primers (5' – 3')

Gene	Sequence forward primer	Sequence reverse primer
HOXB4	TCGAGGTGCCACATATCCAA	TCCCTTGATTCAGCTCACCAA
HOXA6	GGGAGAAAAGTTGGGGAACA	CGCATGAAGTGGAAAAAGGA
HOXA9	CCCCCCCATACACACACTTC	GCCTTCTTGATGGCGTGATT

Table S7: Methylcellulose medium and supplements for CFU assays

Contents	Final concentration	Source
MethoCult SF H4236 Methylcellulose in Iscove's Modified Dulbecco's Media	40%	StemCell Technologies
Fetal Bovine Serum	25%	Gibco, Lot#41Q2105K
Bovine Serum Albumin	2%	PAA
L-Glutamine	2mM	PAA
2-Mercaptoethanol	$5x10^{-5}$ M	PAA
Recombinant Human SCF	50 ng/mL	PAA
Recombinant Human GM-CSF	10 ng/mL	Miltenyi Biotec
Recombinant Human IL-3	10 ng/mL	Miltenyi Biotec
Recombinant Human Epo	3 IU/mL	Invitrogen
Recombinant Human SCF	1,4%	PeproTech

Supplementary References

1. Dahl JA, Collas P. MicroChIP: chromatin immunoprecipitation for small cell numbers. *Methods Mol Biol*. 2009;567:59-74.
2. Li H, Durbin R. Fast and accurate long-read alignment with Burrows-Wheeler transform. *Bioinformatics*. 2010;26(5):589-595.
3. Landt SGea. CHIP-seq guidelines and practices of the ENCODE and modENCODE consortia. *Genome Research*. 2014;22:1813-1831.
4. Zang C, Schones DE, Zeng C, Cui K, Zhao K, Peng W. A clustering approach for identification of enriched domains from histone modification ChIP-Seq data. *Bioinformatics*. 2009;25(15):1952-1958.
5. Heinz S, Benner C, Spann N, et al. Simple combinations of lineage-determining transcription factors prime cis-regulatory elements required for macrophage and B cell identities. *Mol Cell*. 2010;38(4):576-589.
6. Huang da W, Sherman BT, Lempicki RA. Systematic and integrative analysis of large gene lists using DAVID bioinformatics resources. *Nat Protoc*. 2009;4(1):44-57.
7. Shen L, Shao N, Liu X, Nestler E. ngs.plot: Quick mining and visualization of next-generation sequencing data by integrating genomic databases. *BMC Genomics*. 2014;15(1):284.
8. Saeed AI, Sharov V, White J, et al. TM4: a free, open-source system for microarray data management and analysis. *Biotechniques*. 2003;34(2):374-378.
9. Durinck S, Spellman PT, Birney E, Huber W. Mapping identifiers for the integration of genomic datasets with the R/Bioconductor package biomaRt. *Nat Protoc*. 2009;4(8):1184-1191.
